# rBCG-LTAK63 Outperforms BCG in Bladder Cancer Immunotherapy: Dendritic and T Cell Coordination Drives Superior Tumor Control in a Mouse Model

**DOI:** 10.7150/ijbs.118329

**Published:** 2026-01-01

**Authors:** Matheus Ferreira de Almeida, Bruna Gennari Rosa, Daniela Delechiave, Lucas Francisco Annequin, Lazaro Moreira Marques-Neto, Monalisa Martins Trentini, Johanna Christine van Vliet, Isabelle Carolina Cotrim Gozzi, Ana Carolina de Oliveira Carvalho, Dunia Rodriguez, Lennon Ramos Pereira, Giana Carla Gaboardi, Luís Carlos de Souza Ferreira, Luciana Cezar de Cerqueira Leite, Ana Carolina Ramos Moreno

**Affiliations:** 1 Vaccine Development Laboratory, Butantan Institute, São Paulo, SP, Brazil.; 2 Université Claude Bernard Lyon 1, Lyon, France.; 3 Department of Microbiology, Institute of Biomedical Sciences, University of São Paulo, São Paulo, SP, Brazil.

**Keywords:** BCG, recombinant BCG, bladder cancer, immune response, immunotherapy

## Abstract

Bacillus Calmette-Guérin (BCG) remains the standard treatment for non-muscle invasive bladder cancer (NMIBC), yet approximately 30% of patients fail to respond. To enhance therapeutic efficacy, we developed rBCG-LTAK63, a recombinant BCG strain, as a novel immunotherapeutic candidate. *In vitro*, rBCG outperformed BCG in MB49 cell/splenocyte co-cultures by enhancing T cell activation and improving spheroid growth control. *In vivo*, rBCG demonstrated superior antitumor efficacy, significantly reducing the growth of subcutaneously implanted MB49 tumor cells. Immune profiling revealed that rBCG uniquely promoted systemic activation of both CD4⁺ and CD8⁺ T cells, alongside stronger activation of NK and dendritic cells in the spleen. Within the tumor microenvironment, rBCG increased immune cell infiltration, enhanced activation of CD8⁺ T cells and dendritic cells, and decreased the frequency of regulatory T cells, fostering a less immunosuppressive environment. Unlike parental BCG, rBCG-LTAK63 sustained a potent immunostimulatory profile, marked by robust activation of dendritic cells and effector T cells. Similar results were also observed in the orthotopic model, suggesting a translational potential of rBCG-LTAK63. Collectively, our findings demonstrate that rBCG outperforms conventional BCG and represents a promising strategy for improving NMIBC treatment.

## Introduction

Bladder cancer is a malignant neoplasia of the urinary system, accounting for over 90% of all urothelial tumors 1. According to the International Agency for Research on Cancer (IARC), bladder cancer ranks as the eleventh most common malignancy worldwide. In 2022, approximately 600,000 new cases were diagnosed, and more than 220,000 deaths were attributed to this disease globally, emphasizing the critical need for ongoing research in this domain. Upon diagnosis of bladder cancer, it is imperative to ascertain the location and extent of tumor cell growth, particularly whether there is muscular invasion. In its early stages, when confined to the bladder lining, the cancer is classified as non-muscle invasive bladder cancer (NMIBC), comprising about 70% of diagnosed cases 2,3. Conversely, in advanced stages, where cancer penetrates the muscular wall, it is classified as muscle-invasive bladder cancer (MIBC), accounting for 30% of cases 2,3.

Bladder cancer immunotherapy using Bacillus Calmette-Guérin (BCG) has been a pillar of treatment for NMIBC for over four decades, proven to be effective in reducing tumor recurrence and progression in NMIBC 4,5. BCG, a live attenuated strain of *Mycobacterium bovis*, exerts a unique immunomodulatory effect through direct interaction with urothelial and cancer cells, activation of innate immunity, and development of BCG-specific and tumor-specific adaptive immune responses 6,7. The immune responses involve activation and recruitment of various immune cells, including macrophages, neutrophils, dendritic cells and T lymphocytes to the tumor site, promoting a robust antitumor response. The activation of the innate immune responses is a crucial step towards mounting an effector response against the neoplastic cells and is required for the triggering of tumor-specific adaptive responses, which are primarily mediated by antigen-presenting dendritic cells 8. The adaptive responses, in turn, are vital for the control of tumor growth by means of activated tumor-specific cytotoxic T lymphocytes 9.

Despite the efficacy of BCG-based immunotherapy, around 30% of patients do not respond to the treatment, develop resistance, and suffer tumor recurrence 2,10. In recent years, several innovative therapeutic approaches have been explored to overcome BCG failure in NMIBC. For instance, a preclinical study evaluated the intravesical administration of the HER2-targeted antibody-drug conjugate RC48-ADC, demonstrating potent antitumor activity dependent on HER2 expression, significant tumor burden reduction in an orthotopic model, and a favorable safety profile 11. In another approach, a multifunctional nanomedicine (Fe-EGCG@RSL3) was designed to induce ferroptosis in tumor cells and remodel the tumor immune microenvironment, synergizing with antitumor immune responses and achieving superior tumor control in bladder cancer models 12. Together, these studies highlight the need for novel, mechanism-driven interventions capable of enhancing local immune activation and sustaining tumor control.

In this context, recombinant BCG (rBCG) strains emerge as a rational strategy to potentiate intravesical immunotherapy. Our research group has developed a recombinant BCG strain that expresses the non-toxic A subunit of the heat-labile toxin (LTK63) from a mutant strain of enterotoxigenic *Escherichia coli* (rBCG-LTAK63) 13,14. When used prophylactically, rBCG-LTAK63 (rBCG) has proven highly effective in protecting against *Mycobacterium tuberculosis*, demonstrating a reduction in bacillary load in the lungs of infected mice. It induced strong Th1 and Th17 responses, enhanced cytokine expression profiles, and improved innate and long-term immune responses, leading to increased effector and memory T cell responses for up to six months post-immunization 15,16. Additionally, when used therapeutically, the rBCG vaccine was able to induce levels of TGF-β and IL-10 after challenge with *M. tuberculosis*, effectively regulating the intense inflammatory response generated by the pathogen in the lungs 17. Due to its ability to elicit robust immune responses, we believe that rBCG may represent an immunotherapeutic candidate for the treatment of NMIBC.

To investigate the potential tumor control activity exerted by rBCG and determine the associated immune responses, we explored and compared the NMIBC treatments based on BCG or rBCG in a murine heterotopic and orthotopic bladder cancer model. From our results, we demonstrate a superior immunological and therapeutic performance of rBCG regarding BCG for bladder cancer.

## Material and Methods

**Production of working batches of BCG and recombinant BCG.** For all experiments, BCG (Danish) and rBCG-LTAK63 were prepared according to previously described protocols 13. Aliquots of 100 µL were prepared in microtubes and stored at -80 °C. For bacterial concentration assessment via colony-forming units (CFU) counting, we plated 25 µL of diluted bacteria on plates with 7H10 supplemented with oleic acid-albumin-dextrose-catalase (OADC) and 5% glycerol. After 15 to 20 days, colonies were counted to calculate bacterial concentration.

**Animals.** Female C57BL/6 mice (8 weeks old, ~20 g), obtained from the Central Animal Facility of the Butantan Institute, were used in the study. All procedures followed ethical guidelines and were approved by the Institutional Animal Care and Use Committee (CEUA-IB 7301040422 and CEUA-IB 7790070821). Euthanasia was performed via intraperitoneal injection of ketamine (300 mg/kg) and xylazine (30 mg/kg).

**Tumor cell lines.** Murine carcinoma bladder cells MB49, kindly provided by Dr. Yi Lou (University of Iowa, USA), were maintained in RPMI 1640 medium supplemented with 10% fetal bovine serum (FBS) and 1% penicillin/streptomycin (GIBCO - 23400-021) referred to as R10 medium. For the monolayer (2D) and spheroids (3D) cultures, cells were grown in R10 medium further supplemented with 1% sodium pyruvate, essential and non-essential amino acids, L-glutamine, Hepes, and Anti-Anti (referred to as complete medium), under standard conditions (37 °C, 5% CO₂, humidified incubator).

**Splenocytes isolation for *in vitro* assays.** Splenocytes were obtained from mice euthanized with an anesthetic overdose, followed by splenectomy. Spleens were mashed with a sterile 5 mL syringe plunger, suspended in 4 mL RPMI 1640, and filtered through a 40 μm strainer. After centrifugation (5 min, 350 × g), erythrocytes were lysed on ice with lysis buffer. Cells were washed with RPMI, centrifuged, resuspended in complete medium, and counted. Only samples with viability > 85% were used in co-culture and flow cytometry assays.

***In vitro* assay - Bidimensional co-culture (2D).** MB49 cells (1 × 10⁵) were seeded in 24-well plates (500 μL/well, complete medium) and incubated (D0). After 24 h (D1), the medium was replaced with fresh medium containing BCG or rBCG-LTAK63 (MOI 10) and splenocytes from naïve mice (1 × 10⁶; MOI 10), according to the groups: untreated (NT) (MB49 + splenocytes), BCG group (MB49 + splenocytes + BCG), and rBCG group (MB49 + splenocytes + rBCG-LTAK63). Lipopolysaccharide (LPS) at 50 ng/mL was used as a positive control (MB49 + splenocytes + LPS) for immune cell activation Figure S1A). On D2, supernatants were collected and stored at -20 °C for cytokine analysis (BD™ CBA Th1/Th2/Th17 Kit #560485). Cell pellets were stained with antibodies for flow cytometry to assess CD4⁺CD69⁺ and CD8⁺CD69⁺ T cell activation. Antibody details are listed in Table S1. Data were analyzed using FlowJo (Tree Star).

***In vitro* assay - Three-dimensional co-culture (3D).** MB49 cells (1 × 10⁴) were seeded in 96-well Nunclon™ Sphera plates (Thermo Scientific) (200 μL/well, complete medium) for spheroid formation and centrifuged (300 × g, 5 min, 25 °C) before incubation (D0). After 24 h (D1), 150 μL of medium was replaced with fresh medium containing BCG or rBCG-LTAK63 (MOI 50) and/or splenocytes (MOI 10), according to experimental groups (Figure S1B). Lipopolysaccharide (LPS) was used as a positive control (50 ng/mL) for immune cell activation. Subsequently, the cells were incubated. On the subsequent days (D2, D3, and D4), the spheroids were photographed, and the spheroid area was calculated using ImageJ software. On day 4 (D4), the supernatant was stored at -20 °C for cytokine secretion analysis by flow cytometry (BD Cytometric Bead Array (CBA) Mouse Th1/Th2/Th17 Cytokine Kit).

**Heterotopic tumor implantation and immunization protocol.** For tumor implantation, MB49 cells were inoculated at a concentration of 5 x 10^5^ cells/100 μL/animal, in physiological saline solution, into the lower right flank of each mouse via subcutaneous injection on day 0 of the experiment (D0). One day later, on day 1 (D1), immunotherapy cycles with BCG or rBCG-LTAK63 were initiated at a concentration of 8 x 10^6^ CFU, administered by intratumoral route. Then there were three cycles of immunotherapy, with the first treatment on day one (D1), the second treatment on day 8 (D8), and the third treatment on day 15 (D15). Overall, three experimental groups were evaluated: the untreated group (NT) (animals with tumors and no treatment), the BCG group (animals with tumors treated with parental BCG Danish), and the rBCG group (animals with tumors treated with rBCG-LTAK63) (Figure S1C).

**Anesthesia and analgesia for orthotopic tumor model.** Animals were sedated before any invasive procedure by intraperitoneal injection of 50 μL of a mixture of ketamine (50 mg/kg), xylazine (5 mg/kg), and midazolam (2 mg/kg). This procedure induced anesthesia for approximately 60 to 80 minutes, achieving the length and depth of anesthesia necessary for the experimental procedures. When necessary, an anesthetic booster was administered, with a maximum volume of 25 μL. For analgesia, tramadol (40 mg/kg) was administered subcutaneously on the days of invasive procedures. On the remaining days, gabapentin (30 mg/kg) was given orally by gavage every 24 hours to manage pain associated with tumor progression.

**Murine orthotopic tumor implantation model and intravesical immunotherapy.** Animals were sedated as previously described. Once adequate anesthesia was confirmed, the external urogenital area was cleaned with boric acid solution, and transurethral catheterization was performed using a 24-gauge polyethylene catheter lubricated with inert gel (Figure S2A and B). The bladder was emptied and instilled with 100 μL of a 22% ethanol solution, which remained in the bladder for 15 minutes. After this period, the bladder was emptied and flushed twice with 100 μL of PBS to remove residual ethanol. Then, 100 μL of a suspension containing 5 x 10³ MB49 cells in RPMI 1640 medium (Figure S2C) was instilled into the bladder (Figure S2D), where it remained for 1 hour, with body temperature maintained on a heating pad throughout the procedure (Figure S2E). Hematuria was assessed in the following days as an indicator of tumor implantation. One day later, on day 1 (D1), intravesical immunotherapy cycles with BCG or rBCG-LTAK63 were initiated at a concentration of 1 x 10^6^ CFU. Four treatment cycles were administered at 7-day intervals (D1, D8, D15, and D22). On each treatment day, animals were anesthetized and transurethrally catheterized as previously described. After the bladder was emptied, 100 μL of either sterile saline or a saline suspension containing BCG or rBCG was instilled and retained in the bladder for 1 hour. On day 25 (D25), animals were euthanized as scheduled for experimental endpoint assessment. Bladders and spleens were harvested and processed to obtain total immune cells.

**Evaluation of tumor volume and mice survival.** Tumor progression in the heterotopic model was measured twice weekly for up to 22 days, while mice survival was monitored for up to 50 days in both heterotopic and orthotopic tumor models. Tumor volume was calculated using the formula "(largest diameter x smallest diameter^2^) / 2" with tumor diameter data obtained using a caliper. Humanized euthanasia was performed when the mouse's tumor volume reached 1000 mm³ or when the animal exhibited one or more signs of pain, according to the Grimace Scale. During survival assays, animals were monitored for body weight and were euthanized if they lost more than 20% of their initial weight. In the orthotopic model, tumor burden was assessed on day 25 (D25) following euthanasia.

**Immunophenotyping assay and microenvironment analyses.** To evaluate the activation of the systemic (only for the heterotopic model) response 7 days after the first cycle of immunotherapy (D8) and 7 days after the last immunotherapy (Day 22 - D22), 6-8 drops of peripheral blood were collected via submandibular puncture, for analysis of CD4 and CD8 T lymphocyte activation. Importantly, this procedure was not performed in animals from the orthotopic model, as these animals naturally present hematuria due to tumor development, and the intervention could negatively impact both the experimental outcome and animal survival. On D22 (heterotopic model) or D25 (orthotopic model), all mice were euthanized, followed by removal of tumors and spleens, which were processed to obtain total immune cells. These cells were collected and processed according to standard protocols previously described (18,19. The immune microenvironment of these organs was immunophenotyped and evaluated for the frequency and activation of CD4 and CD8 T lymphocytes, frequency and activation of myeloid cells, macrophages, neutrophils, dendritic cells, frequency and activation of NK cells, and frequency of regulatory T cells. Antibodies used are displayed on Table S1.

**Intracellular cytokine staining.** The cells obtained from the processing of the spleen and the tumor were stimulated for the lymphocyte panel with 50 μL of complete medium containing the stimuli (0.5 μL of CD28 and 1 μL of CD3 per well) were added. For the myeloid panel, 50 μL of complete medium without stimulus was added. After 4 to 6 hours, 50 μL of complete medium containing brefeldin A (0.12 μL per well) was added, and the samples were incubated for up to 12 hours in an incubator at 37°C with 5% CO_2_. The marking protocol was then followed. Flow cytometry was executed using BD FACS Canto II and analyzed using FlowJo software (BD Biosciences). On the day of immunophenotyping of immune cells, the extracellular antibody mixes and FMOs (fluorophore minus one) were prepared in PBS containing 2% FBS. Simultaneously, the intracellular antibody mix was prepared in Perm/Wash buffer (BD) and kept protected from light in the refrigerator. The labeling protocol used was established by Pagni LR, et al. 2022 19. Antibodies used are displayed on Table S1.

**Correlation analysis of immune cells data in relation to tumor volume.** The correlogram was generated using SRplot (bioinformatics.com.cns). Spearman's correlation was utilized to determine the link between the clinical and immunological variables assessed on day 20 post-treatment. The significance level was established at P < 0.05.

**Statistical analysis.** The GraphPad-Prism program (GraphPad Software, San Diego, CA, USA) was used for the validation of the results, and the unpaired T-test, One-Way ANOVA, or Two-Way ANOVA were adopted. The results were confirmed through multiple comparisons using Tukey's test. Survival curves were generated using the Kaplan-Meier method and compared using the log-rank (Mantel-Cox) test. Values of p < 0.05 were considered significant.

## Results

### rBCG Improves CD4 and CD8 T Lymphocyte Activation in a Bidimensional Co-culture with MB49 Cells

To assess the inflammatory potential of rBCG, we performed a co-culture assay of MB49 bladder carcinoma cells with splenocytes. Cytokine production was analyzed to evaluate Th1, Th2, and Th17 responses, alongside CD69 expression as a marker of T cell activation. Cytokine analysis of the culture supernatant Figure S3 revealed a significant increase in pro-inflammatory cytokine production following stimulation with either BCG or rBCG at MOI 10 as compared to the untreated (NT) group Figure S3B-F). LPS was used as a positive control (Figure S4. Notably, rBCG induced significantly higher levels of TNF-α by splenocytes compared to BCG Figure S3F), suggesting an enhanced inflammatory response. Interestingly, rBCG also promoted a significant increase in IL-10 production (Figure S3C), an anti-inflammatory cytokine, indicating more complex immune modulation. Regarding T cell activation, rBCG induced a significantly higher activation of both CD4⁺ and CD8⁺ T cells compared to both the NT and BCG groups (Figure S3H). These findings indicate that, *in vitro*, rBCG exhibits an enhanced capacity to activate T cells while driving a stronger pro-inflammatory cytokine response compared to parental BCG.

### BCG and rBCG Suppress MB49 Spheroid Growth, with Enhanced Efficacy in the Presence of Immune Cells

**Figure 1:**
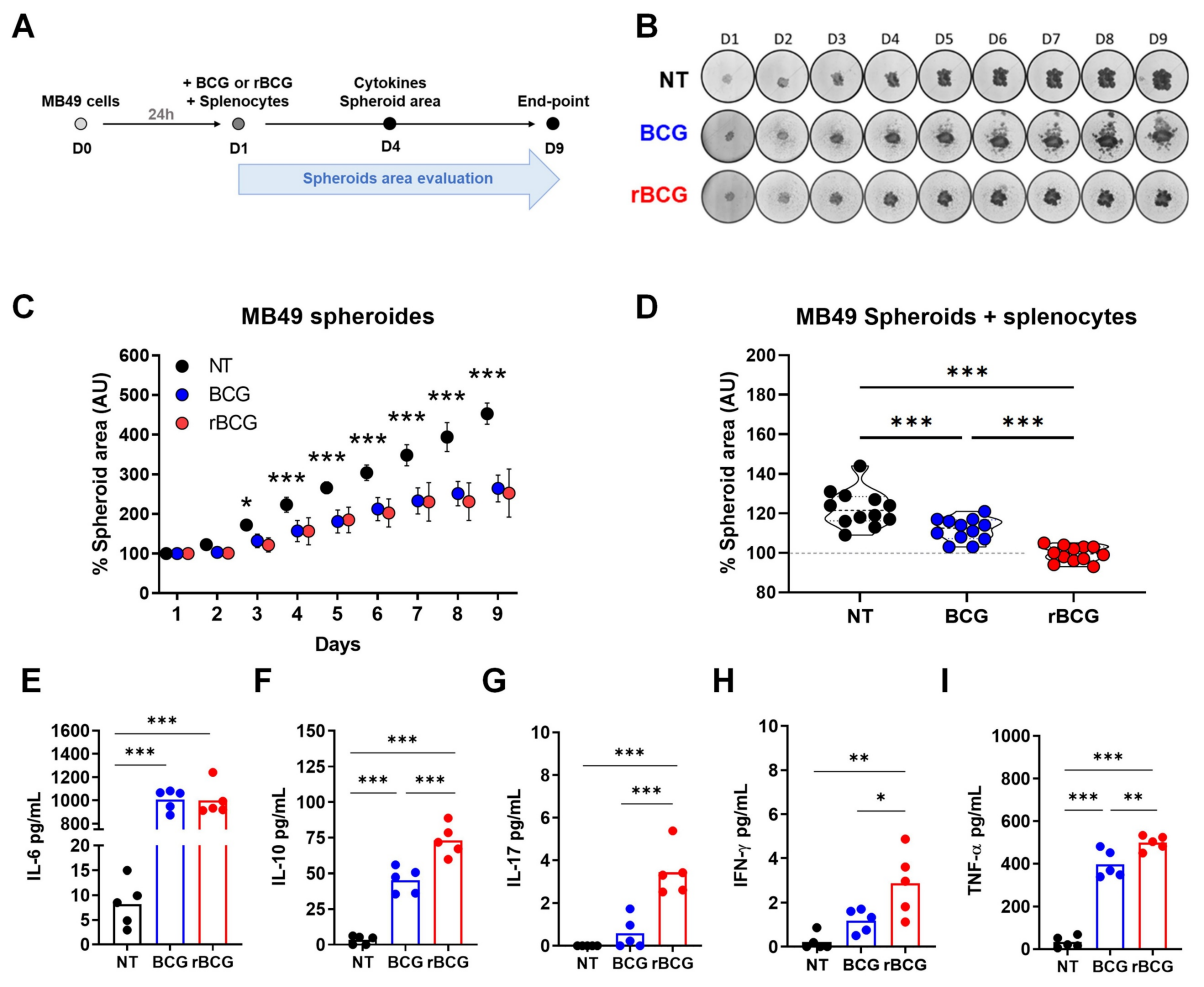
** The inflammatory potential of rBCG contributes to better control of tumor spheroids in the presence of immune cells.** (A) Timeline of the experimental design. (B) Images of spheroids from each experimental group over 9 days. (C) Graph of spheroid area measured over 9 days. (D) Graph of spheroid area, measured over 4 days. (E) Levels of IL-6 in the co-culture supernatant. (F) Levels of IL-10 in the co-culture supernatant. (G) Levels of IL-17 in the co-culture supernatant. (H) Levels of IFN-γ in the co-culture supernatant. (I) Levels of TNF-α in the co-culture supernatant. D1 - day 1; D2 - day 2; D3 - day 3; D4 - day 4; D5 - day 5; D6 - day 6; D7 - day 7; D8 - day 8; D9 - day 9; NT - untreated; BCG - BCG Danish; rBCG - rBCG-LTAK63; AU - arbitrary units. ANOVA statistical analysis (*p < 0.05, ***p < 0.001).

BCG-based immunotherapy exerts antitumor effects through direct tumor cell infection and cytotoxicity, alongside immune-mediated mechanisms. To evaluate this effect, we established a three-dimensional spheroid culture model, exposing MB49 spheroids to BCG or rBCG at MOI 50, in the presence or absence of immune cells (Figure 1A). The analysis of spheroid growth inhibition revealed that both BCG and rBCG effectively suppressed spheroid expansion starting on day 3 of the experiment (Figures 1B and C). Tumor volume was monitored for nine days, and no significant differences were observed between BCG and rBCG in the absence of immune cells. Nonetheless, when immune cells were introduced to the spheroid microenvironment, both BCG and rBCG significantly inhibited spheroid growth compared to the untreated (NT) group. rBCG exhibited superior tumor growth suppression compared to BCG, underscoring the crucial role of immune activation in rBCG antitumor effects (Figure 1D).

To further characterize the immunological microenvironment of co-cultured MB49 spheroids, we analyzed cytokine secretion profiles. Both BCG and rBCG induced IL-6 production with significant difference compared to NT (Figure 1E). Remarkably, in contrast to the two-dimensional co-culture model, only rBCG induced significant secretion of IL-17 (Figure 1G) and IFN-γ (Figure 1H). Consistent with the bidimensional model, rBCG promoted higher TNF-α (Figure 1I) and IL-10 (Figure 1F) production than BCG, reinforcing the enhanced immunostimulatory profile. This cytokine pattern aligns with the findings from the two-dimensional co-culture, reaffirming that rBCG elicits a stronger pro-inflammatory response than the parental BCG.

### rBCG Demonstrates Superior Immunotherapeutic Efficacy Compared to Parental BCG in the Control of Subcutaneous Urothelial Tumors in Mice

**Figure 2:**
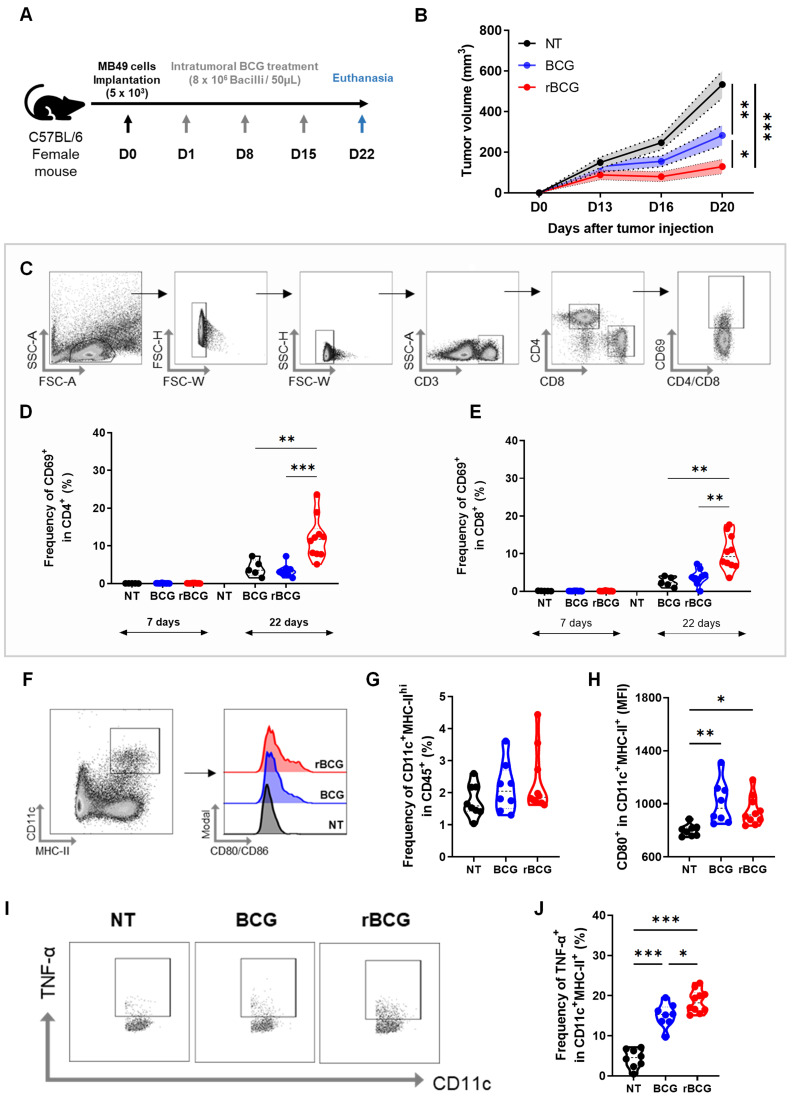
** rBCG shows superior immunotherapeutic efficacy over parental BCG in controlling subcutaneous urothelial tumors and enhances splenic dendritic cell activation.** (A) Experimental design of the *in vivo* trial, indicating the days of intratumoral/peritumoral immunotherapy cycles. (B) Tumor volume graph. (C) Strategy for analysis used to separate CD4^+^ and CD8^+^ T cell populations and their respective CD69 expression. (D) CD69 expression graph within CD4 at D7 (Day 7) and D22 (Day 22). (E) CD69 expression graph within CD8 at D7 (Day 7) and D22 (Day 22). (F) Analysis strategy for dendritic cell panel. CD45-expressing cells were selected from the previous single-cell analysis. (G) Frequency of CD11c^+^ MHC-II^+^ population. (H) Median fluorescence of CD80 within the CD11c^+^ MHC-II^+^ population. (I) Analysis strategy for the dendritic cell panel regarding TNF-α production. (J) Frequency of TNF-α expression within the CD11c^+^ MHC-II^+^ cell population. D0 - day zero, D1 - day 1; D8 - day 8; D15 - day 15; D22 - day 22; NT - no treated; BCG - BCG Danish; rBCG - rBCG-LTAK63. ANOVA statistical analysis (*p < 0.05, **p < 0.01, ***p < 0.001). Data are representative of two independent experiments, with n=10 mice per experimental group.

After confirming that rBCG elicits stronger immune activation and superior *in vitro* tumor spheroid suppression compared to parental BCG, we next evaluated its antitumor efficacy in an experimental bladder cancer model. For this, we employed a subcutaneous tumor implantation model (Figure 2A). Treatment with BCG resulted in a significant reduction in tumor volume compared to the untreated (NT) group (Figure 2B), consistent with its established role as the standard therapy for NMIBC. Notably, by day 20 (D20), rBCG exhibited a greater reduction in tumor volume compared to BCG, indicating enhanced therapeutic activity.

To further characterize the systemic immune activation, we analyzed CD4⁺ and CD8⁺ T cell activation profiles six days after the first immunotherapy cycle (D7) and seven days after the final treatment cycle (D22). Blood samples were collected, and CD69 expression was assessed as a marker of T cell activation (Figure 2C). On D7, no significant activation of CD4⁺ (Figure 2D) or CD8⁺ T cells (Figure 2E) was observed in any experimental group. On the other hand, by D22, rBCG was the only treatment capable of significantly activating both CD4⁺ and CD8⁺ T cells, demonstrating a more robust and sustained systemic immune response compared to both NT and BCG groups.

### Compared to Parental BCG, rBCG Induces Increased Activation of Innate and Adaptive Immune Cells in the Spleen of Tumor-Bearing Mice

On D22, following euthanasia, splenic immune cells were immunophenotyped to assess innate and adaptive immune activation. In the myeloid cell panel (Figure S5A), both BCG and rBCG increased the frequency of inflammatory monocytes (Figure S5B), resident monocytes (Figure S5C), and neutrophils (Figure S5D) compared to the NT group. Notably, rBCG induced a greater recruitment of neutrophils than parental BCG. The activation profiles of these cells were largely similar among the groups (Figure S5E, F and G), except for resident monocytes, which exhibited significantly higher activation following both BCG and rBCG treatment.

Regarding splenic dendritic cell populations, no significant differences were found in their frequency among groups (Figure 2F and G). However, dendritic cells from both BCG- and rBCG-treated animals exhibited greater activation, as indicated by increased CD80 expression (Figure 2H). Additionally, both BCG and rBCG promoted higher TNF-α production compared to the NT group (Figure 2I), with rBCG inducing significantly higher levels than parental BCG (Figure 2J). These results suggest that rBCG triggers a more pronounced inflammatory response, further supporting its superior immunostimulatory potential.

**Figure 3:**
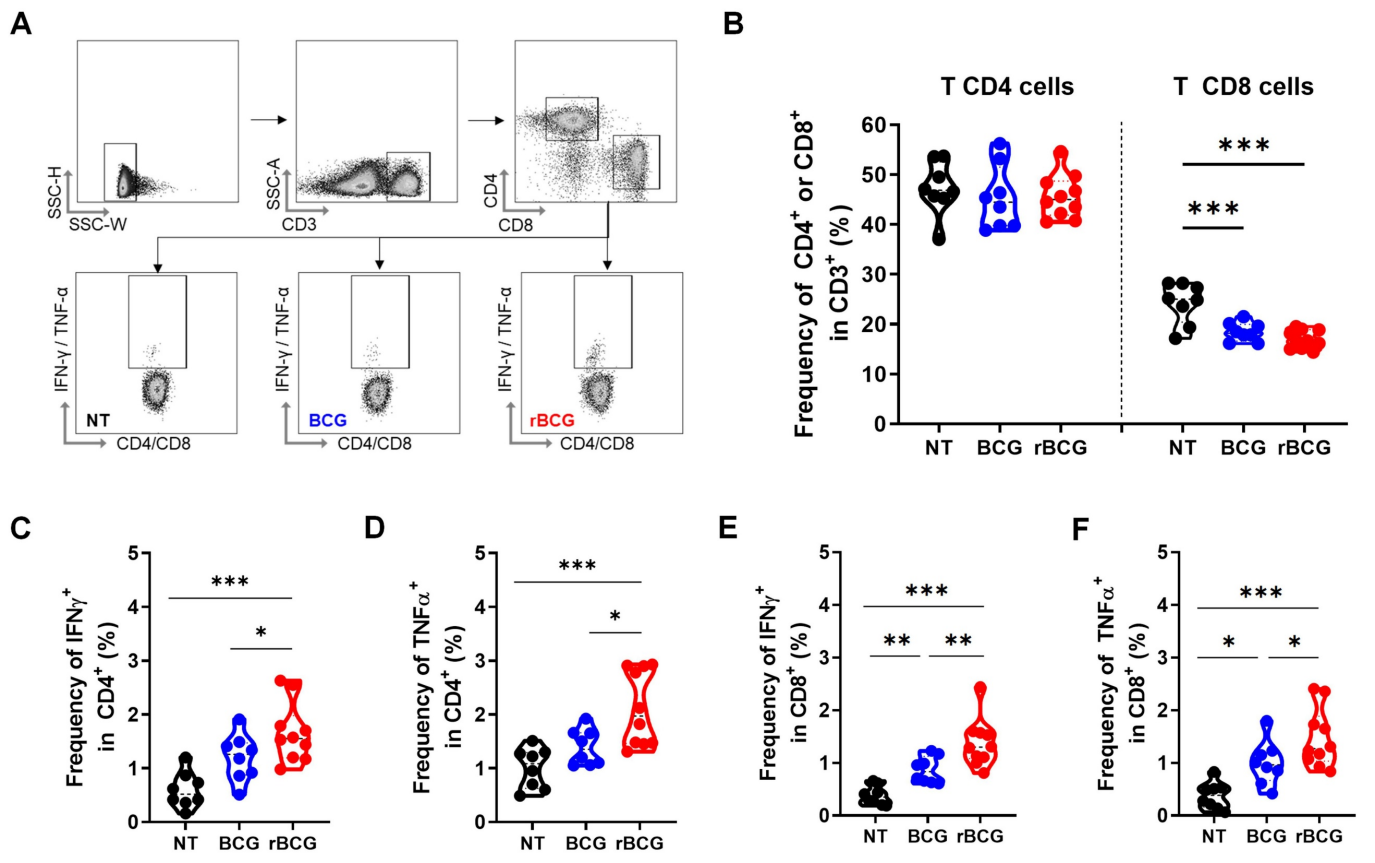
** rBCG treatment enhances T cell activation in the spleen.** (A) Gating strategy for T cell panel. (B) Frequency of CD4⁺ and CD8⁺ T cells. (C) Frequency of IFN-γ⁺ cells within the CD4⁺ T cell population. (D) Frequency of TNF-α⁺ cells within the CD4⁺ T cell population. (E) Frequency of IFN-γ⁺ cells within the CD8⁺ T cell population. (F) Frequency of TNF-α⁺ cells within the CD8⁺ T cell population. NT - untreated; BCG - parental BCG (Danish); rBCG - rBCG-LTAK63. Statistical analysis performed using ANOVA (*p < 0.05, **p < 0.01, ***p < 0.001). Data are representative of two independent experiments, with n=10 mice per experimental group.

In the splenic T lymphocyte analysis (Figure 3A), no significant differences were observed in the frequency of CD4⁺ T cells across groups (Figure 3B). However, rBCG induced a higher activation profile, as evidenced by the increased frequency of IFN-γ⁺ (Figure 3C) and TNF-α⁺ (Figure 3D) CD4⁺ T cells, surpassing both BCG and NT groups. Notably, no significant differences were detected between the NT and BCG groups. Regarding CD8⁺ T cells, the overall frequency was higher in the NT group compared to both treated groups (Figure 3B). Nonetheless, the functional capacity of CD8⁺ T cells was significantly higher after the immunotherapy, with higher IFN-γ (Figure 3E) and TNF-α (Figure 3F) production in the BCG-treated groups. Importantly, rBCG elicited a stronger cytokine response than parental BCG, reinforcing the superior ability to promote robust adaptive immune activation.

A similar pattern was observed in the analysis of NK cells (Figure S6A), where no significant differences were detected in the NK cell frequencies among the three groups (Figure S6B). However, rBCG induced significantly higher IFN-γ production compared to both BCG and NT groups. Additionally, TNF-α levels were elevated in both BCG- and rBCG-treated groups relative to NT, highlighting a higher inflammatory response following the immunotherapy.

To further characterize NK cell activation and differentiation, we assessed their maturation profile (Figure S6C). The frequency of immature NK cells (CD27⁺CD11b⁻) remained unchanged across groups, whereas inflammatory NK cells (CD27⁺CD11b⁺) were more prevalent in the BCG-treated group compared to the other conditions. The proportion of mature NK cells (CD27⁻CD11b⁺) was consistent among all groups, but expression of KLRG, a marker of terminal activation/maturation, was significantly higher in both BCG-treated groups compared to NT. These findings suggest that both BCG and rBCG enhance NK cell functionality, driving an inflammatory cytokine response while promoting advanced NK cell maturation.

### rBCG Enhances Innate and Adaptive Immune Cell Activation in the Tumor Microenvironment Compared to Parental BCG

**Figure 4:**
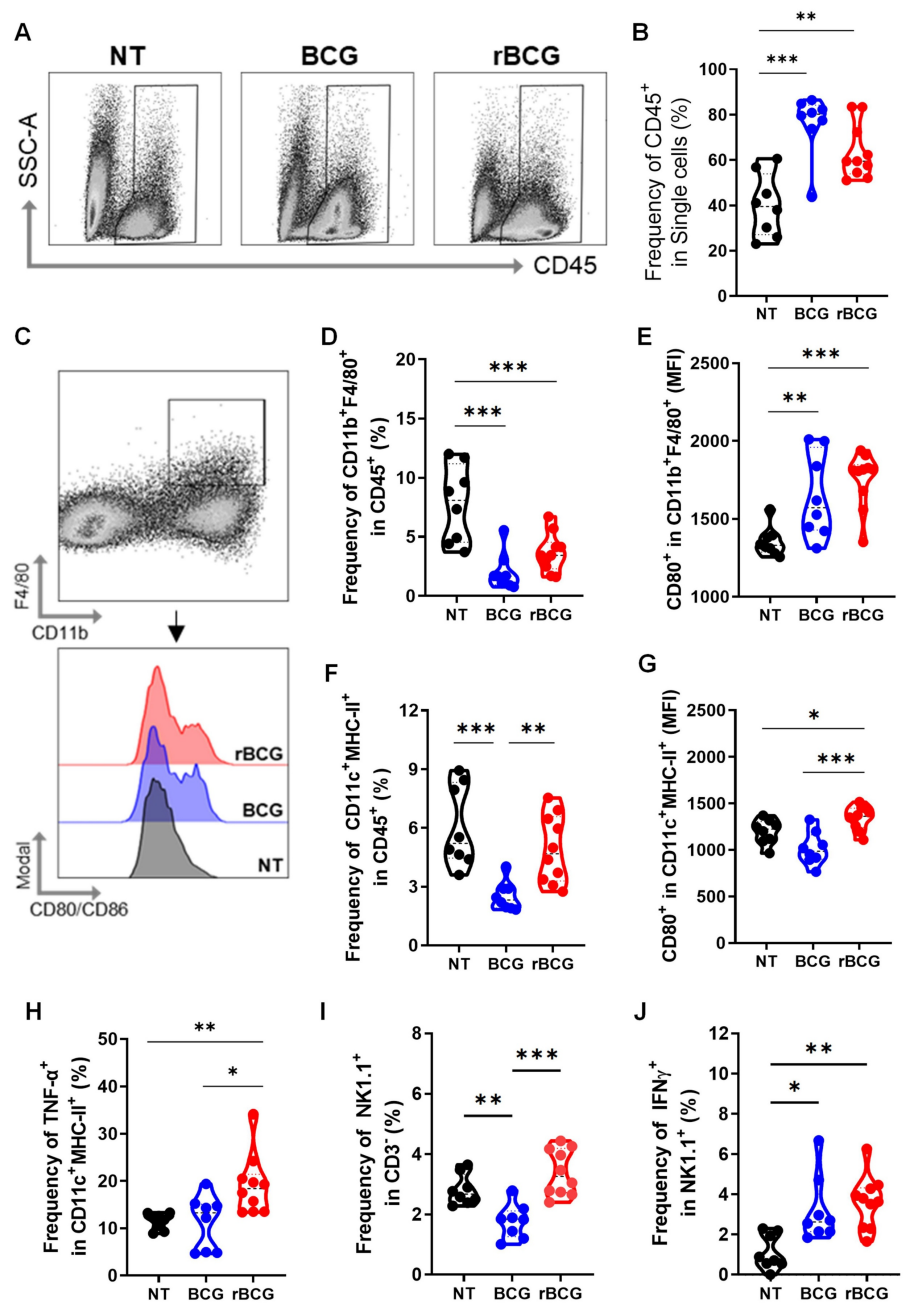
** rBCG induces greater activation of innate immune cells in the tumor microenvironment.** (A) Gating strategy for CD45-expressing cells. (B) Frequency of CD45^+^ cell population. (C) Gating strategy for macrophages panel. (D) Frequency of F4/80^+^ CD11b^+^ population. (E) Median fluorescence of CD80 within the F4/80^+^ CD11b^+^ population. (F) Frequency of MHC-II^+^ CD11c^+^ population. (G) Median fluorescence of CD80 within the MHC-II^+^ CD11c^+^ population. (H) Frequency of TNF-α expression within the MHC-II^+^ CD11c^+^ population. (I) Frequency of NK1.1^+^ population. (J) Frequency of IFN-γ expression within the NK1.1^+^ population. NT - no treated; BCG - BCG Danish; rBCG - rBCG-LTAK63. ANOVA statistical analysis (*p < 0.05, **p < 0.01, ***p < 0.001). Data are representative of two independent experiments, with n=10 mice per experimental group.

To assess the impact of immunotherapy on the tumor immune microenvironment, we evaluated multiple immunological parameters. Both BCG and rBCG treatments increased immune cell infiltration, as indicated by the elevated expression of CD45⁺ cells within the tumor microenvironment (Figures 4A and B). Regarding tumor-infiltrating macrophages (Figure 4C), the frequencies were significantly higher in the NT group compared to both treated groups (Figure 4D). However, macrophage activation, assessed by CD80 expression, was significantly higher in the BCG- and rBCG-treated groups compared to NT (Figure 4E), indicating enhanced immune activation despite lower macrophage infiltration. For intratumoral dendritic cells, the frequencies of these cells were similar between the NT and rBCG groups, while BCG treatment resulted in a significantly lower frequency (Figure 4F). Importantly, rBCG exhibited superior dendritic cell activation, as evidenced by increased CD80 expression (Figure 4G) and TNF-α production (Figure 4H). This activation was significantly higher compared to both the NT and BCG groups, which showed no significant difference between them.

Analysis of tumor-infiltrating NK cells revealed that higher frequencies were found in the NT and rBCG groups regarding the BCG group (Figure 4I). However, both BCG and rBCG significantly increased IFN-γ production relative to NT (Figure 4J), indicating enhanced functional activation of NK cells. These findings demonstrate that while both BCG and rBCG effectively activate intratumoral innate immune responses, only rBCG can stimulate dendritic cells within this microenvironment. This finding highlights dendritic cells as a key immunological differentiator between BCG and rBCG, further supporting the enhanced immunotherapeutic potential of rBCG.

**Figure 5:**
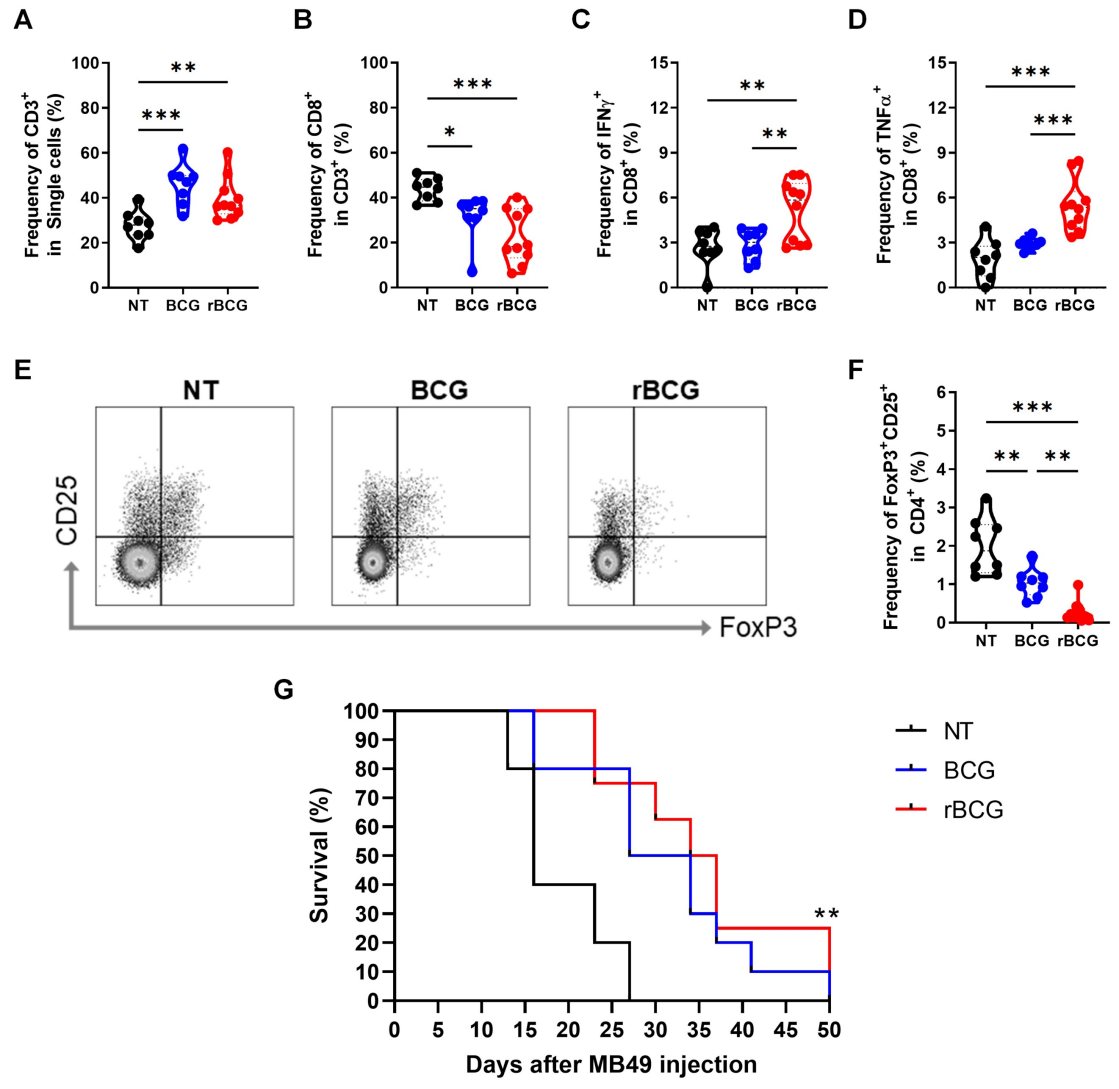
** rBCG Enhances CD8⁺ T Cell activation and mitigates immunosuppression in the tumor microenvironment.** (A) Frequency of CD3^+^ population. (B) Frequency of CD8^+^ population. (C) Frequency of IFN-γ expression within the CD8^+^ population. (D) Frequency of TNF-α expression within the CD8^+^ population. (E) Gate strategy of Treg cells population. (F) Frequency of CD25^+^ FoxP3^+^ population. (G) Kaplan-Meier survival curves of tumor-bearing mice (data are representative of one experiment, with n=10 mice per experimental group). Statistical significance indicates comparisons between the untreated group and both treated groups. NT - no treated; BCG - BCG Danish; rBCG - rBCG-LTAK63. ANOVA statistical analysis (*p < 0.05, **p < 0.01, ***p < 0.001). Data are representative of two independent experiments, with n=10 mice per experimental group.

Analysis of tumor-infiltrating lymphocytes revealed that both BCG and rBCG treatments significantly increased the frequency of CD3⁺ T cells compared to the NT group (Figure 5A). Interestingly, although the frequency of CD8⁺ T cells was lower in both BCG-treated groups compared to NT (Figure 5B), only rBCG was capable of effectively activating CD8⁺ T cells, as demonstrated by the higher induction of IFN-γ (Figure 5C) and TNF-α (Figure 5D). Notably, no significant differences in CD8⁺ T cell activation were observed between the NT and BCG groups, highlighting rBCG's unique ability to enhance CD8⁺ T cell function. To further evaluate the immunosuppressive landscape within the tumor microenvironment, we analyzed FoxP3 and CD25 expression in CD4⁺ T cells (Figure 5E). The NT group exhibited the highest levels of these immunosuppressive markers, followed by a moderate reduction in the BCG-treated group, with rBCG treatment inducing the most pronounced decrease in expression (Figure 5F). These findings emphasize the superior immunotherapeutic potential of rBCG, as it effectively activates CD8⁺ T cells and mitigates regulatory T cell-mediated immunosuppression, fostering a more favorable antitumor immune response compared to parental BCG. When evaluating animal survival over an extended period, both BCG and rBCG treatments significantly prolonged survival compared with the untreated group, with a clear trend toward improved survival in rBCG-treated mice (Figure 5G). These results indicate that rBCG offers a more consistent survival benefit in the subcutaneous bladder cancer model, confirming its improved therapeutic performance over parental BCG.

### The Immune Triad of CD4⁺ T Cells, CD8⁺ T Cells, and Dendritic Cells Correlates with Enhanced Tumor Control

**Figure 6:**
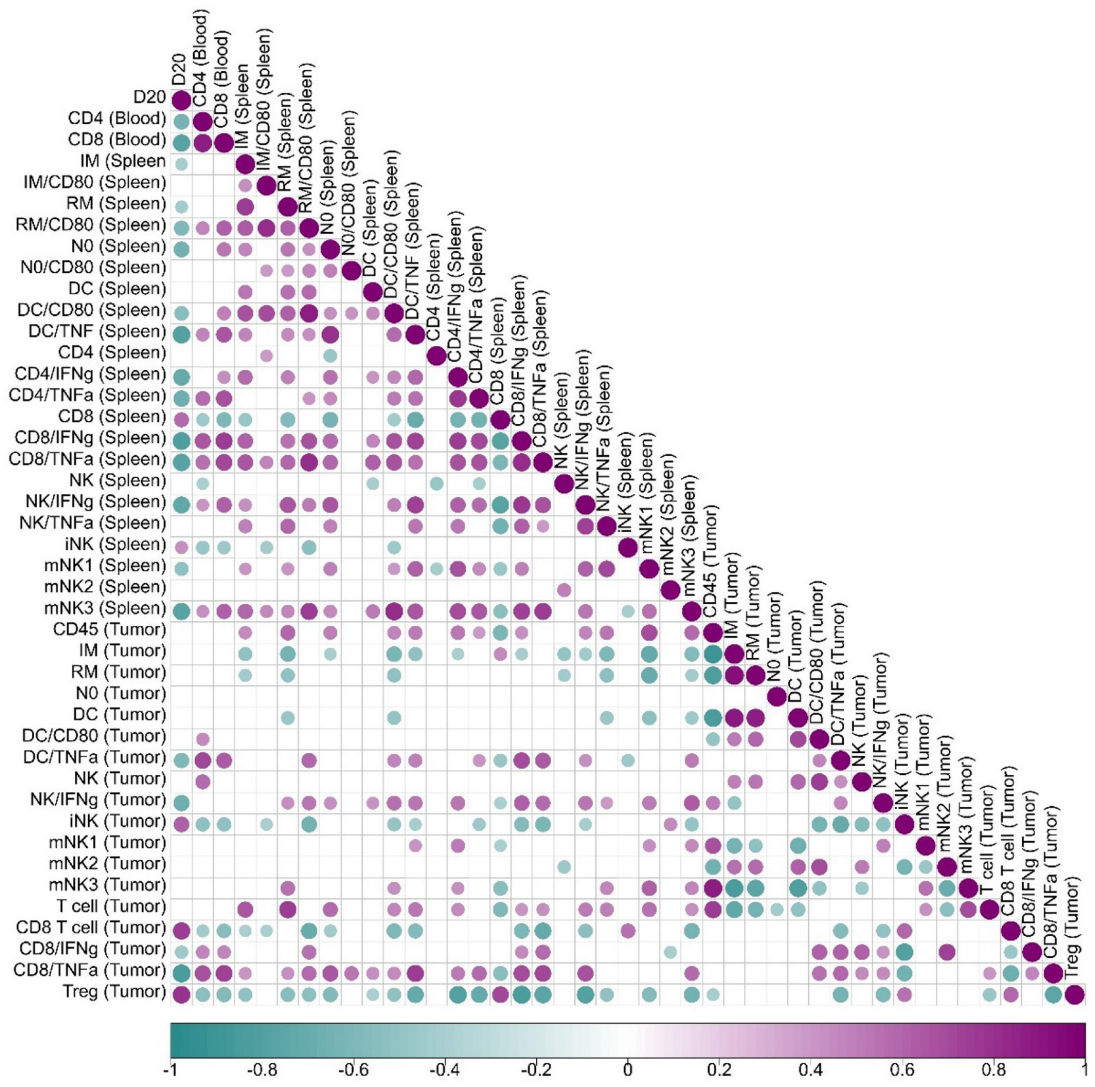
** Correlation analysis of immune cells data in relation to tumor volume.** Spearman's correlation was employed to evaluate the level of association between each pair of clinical or biological characteristics observed in mice. Positive correlations are represented in violet, whereas negative correlations are depicted in teal. The color intensity and dimensions of the circles correspond to the correlation coefficients. The legend at the bottom of the correlogram displays the correlation coefficients together with their related colors. Data displays only correlation with p < 0.05.

A correlation analysis between immune cell activation and tumor volume progression (Figure 6 revealed key associations that define the immunological landscape of tumor control. In peripheral blood, mice exhibiting higher activation of CD4⁺ and CD8⁺ T cells displayed significantly smaller tumor volumes, establishing a strong inverse correlation between T cell activation and tumor growth. This phenomenon was further correlated with heightened dendritic cell activity within the tumor microenvironment, which was linked to increased TNF-α production, suggesting a positive feedback loop where CD4⁺ T cells, CD8⁺ T cells, and dendritic cells mutually enhance each other's activation, thereby amplifying the antitumor immune responses.

In the spleen, increased frequencies of neutrophils, activated resident monocytes, and inflammatory monocytes were associated with superior tumor control. Notably, TNF-α producing activated dendritic cells (CD80⁺) correlated with enhanced tumor suppression. Similarly, CD4⁺ and CD8⁺ T cells producing IFN-γ and TNF-α were positively associated with improved tumor control, alongside mature NK cells producing IFN-γ, whose elevated presence further supported antitumor immunity. CD4⁺ T cell activation in the spleen was directly linked to increased IFN-γ and TNF-α production by CD8⁺ T cells within the tumor microenvironment, fostering a reduction in Tregs and mitigating local immunosuppression.

Within the tumor microenvironment, effective tumor control was strongly associated with higher frequencies of TNF-α-producing dendritic cells, IFN-γ-producing NK cells, and CD8⁺ T cells secreting both IFN-γ and TNF-α. Importantly, activated dendritic cells producing TNF-α correlated with increased tumor-infiltrating T cell frequencies, heightened TNF-α production by CD8⁺ T cells, and a concurrent reduction in Tregs, further contributing to a less immunosuppressive tumor milieu. Conversely, the presence of Tregs strongly correlated with tumor growth, serving as a marker of poor prognosis.

These findings emphasize the critical role of the coordinated activation of CD4⁺ T cells, CD8⁺ T cells, and dendritic cells in mediating effective tumor control. Enhanced activation of this immune triad was associated with superior tumor suppression and a significant reduction in Tregs, highlighting the synergy between these immune cell populations in orchestrating a potent antitumor response. The correlation analysis and the immunological and clinical outcomes of rBCG immunotherapy underscores the pivotal role of orchestrated interactions between CD4⁺ T cells, CD8⁺ T cells, and dendritic cells in shaping the tumor microenvironment.

### rBCG Demonstrates Superior Control of Tumor Growth in the Orthotopic Bladder Cancer Model

**Figure 7:**
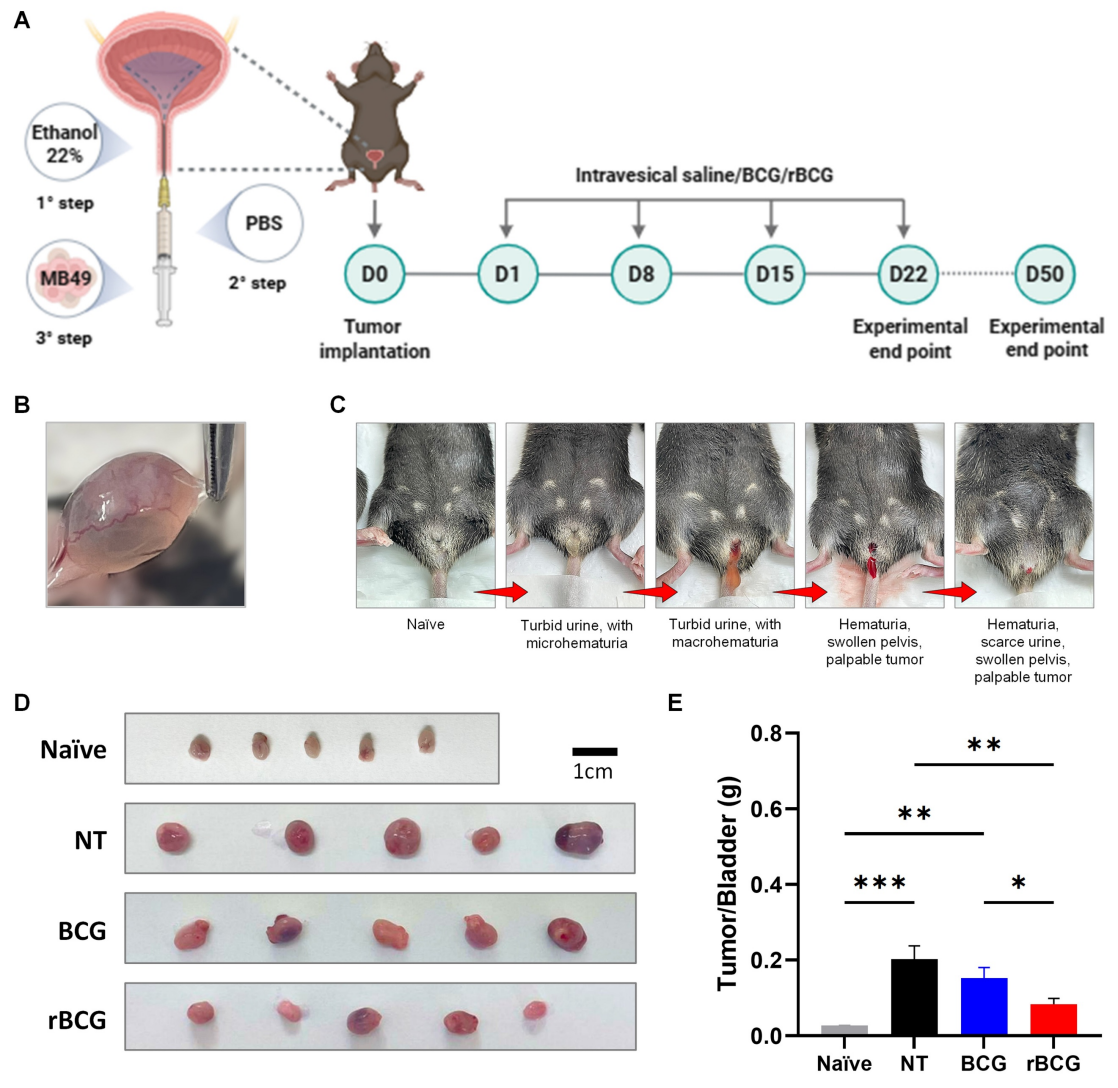
** rBCG demonstrated superior immunotherapeutic efficacy compared to parental BCG in the control of orthotopic bladder tumor in mice.** (A) Experimental design of the *in vivo* trial, showing the timeline of tumor implantation and intravesical immunotherapy cycles. (B) Representative macroscopic image of tumor formation in the bladder. (C) Clinical signs of tumor burden in the orthotopic bladder cancer model, highlighting hematuria as the indicator of tumor progression. (D) Representative images of the bladders from the experimental groups. (E) Tumor/Bladder weight graph. NT - no treated; BCG - BCG Danish; rBCG - rBCG-LTAK63. ANOVA statistical analysis (*p < 0.05, **p < 0.01, ***p < 0.001). Data are representative of two independent experiments, with n=10 mice per experimental group.

In the orthotopic bladder cancer model, intravesical immunotherapy with rBCG demonstrated superior tumor control compared with parental BCG. As shown in the experimental design (Figure 7A), mice underwent a three-step tumor implantation procedure followed by four weekly intravesical instillations of saline, BCG, or rBCG. Macroscopic examination confirmed successful tumor formation in the bladder (Figure 7B). Clinical monitoring revealed progressive disease characterized by turbid urine with micro- and macrohematuria, followed by visible hematuria and pelvic swelling as tumor burden increased (Figure 7C). At the experimental endpoint (D25), bladders were excised and weighed, revealing a marked reduction in tumor mass in both BCG- and rBCG-treated animals compared with untreated controls (Figure 7D). Notably, rBCG-treated mice exhibited the lowest tumor/bladder weight ratio, indicating enhanced therapeutic efficacy relative to parental BCG (Figure 7E).

### rBCG Potentiates Antigen-Presenting Cell Activation, Promotes Effector Cell Responses, and Reduces Spleen- and Tumor-Associated Immunosuppression

**Figure 8:**
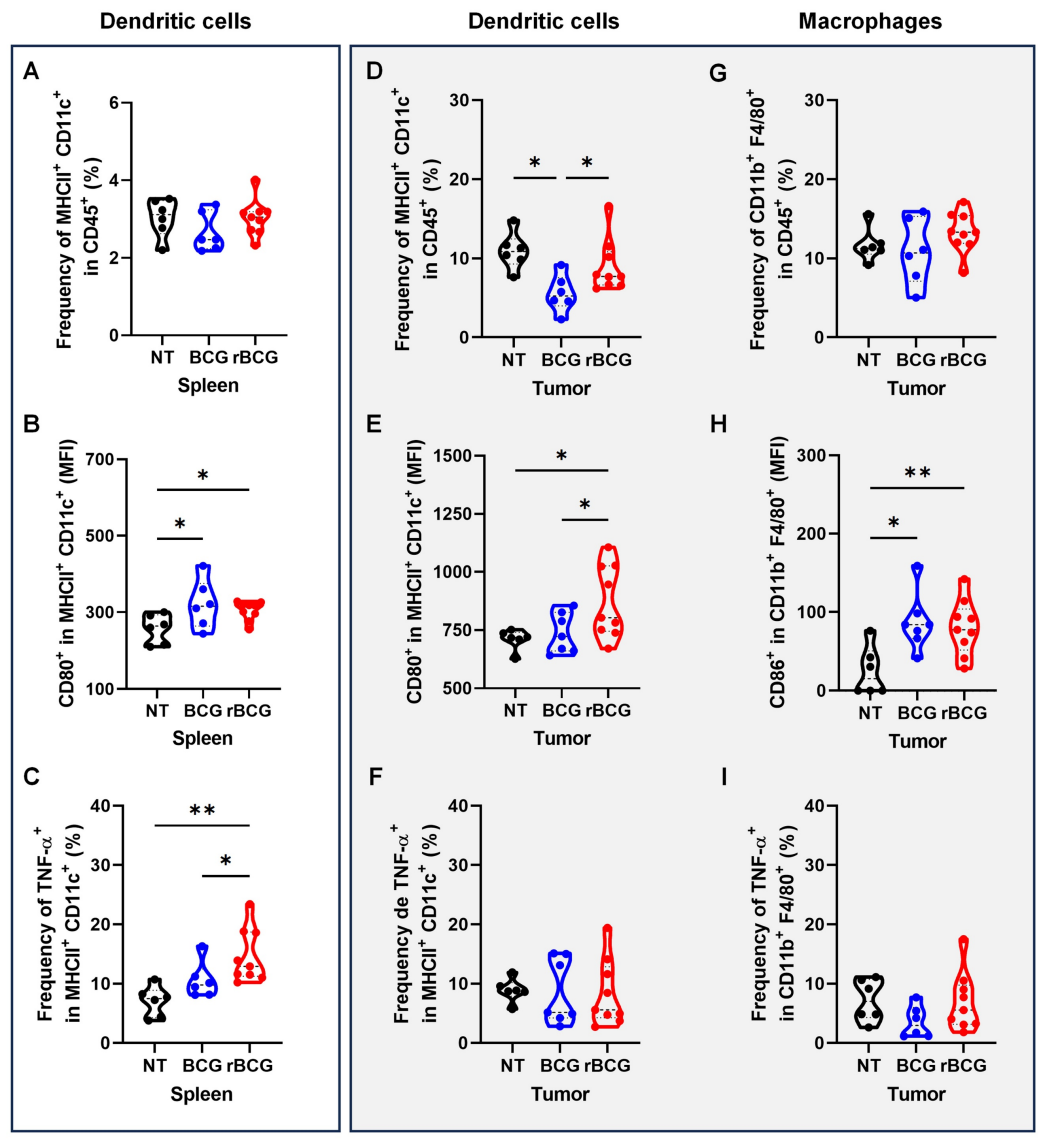
** rBCG induces greater activation of dendritic cells in spleen and in the tumor microenvironment.** (A) Frequency of MHC-II^+^ CD11c^+^ population in the spleen. (B) Median fluorescence of CD80 within the MHC-II^+^ CD11c^+^ population in the spleen. (C) Frequency of TNF-α expression within the MHC-II^+^ CD11c^+^ population in the spleen. (D) Frequency of MHC-II^+^ CD11c^+^ population in the tumor. (E) Median fluorescence of CD80 within the MHC-II^+^ CD11c^+^ population in the tumor. (F) Frequency of TNF-α expression within the MHC-II^+^CD11c^+^ population in the tumor. (G) Frequency of F4/80^+^ CD11b^+^ population in the tumor. (H) Median fluorescence of CD86 within the F4/80^+^ CD11b^+^ population in the tumor. (I). Frequency of TNF-α expression within the F4/80^+^ CD11b^+^ population. NT - no treated; BCG - BCG Danish; rBCG - rBCG-LTAK63. ANOVA statistical analysis (*p < 0.05, **p < 0.01, ***p < 0.001). Data are representative of two independent experiments, with n=10 mice per experimental group.

Analysis of antigen-presenting cells revealed that rBCG treatment induced a more pronounced activation of dendritic cells both systemically and within the tumor microenvironment. In the spleen, rBCG did not significantly alter the frequency of MHC-II⁺ CD11c⁺ cells compared to NT and BCG (Figure 8A), but significantly increased CD80 expression (Figure 8B) and TNF-α production (Figure 8C), indicating enhanced maturation and activation status. Within the tumor, rBCG promoted an increased frequency of MHC-II⁺ CD11c⁺ dendritic cells compared to both NT and BCG groups (Figure 8D), accompanied by higher CD80 expression (Figure 8E). Although TNF-α production by intratumoral dendritic cells showed no major differences (Figure 8F), rBCG-treated mice exhibited significantly elevated CD86 expression in tumor-associated macrophages (Figure 8H), suggesting improved costimulatory capacity within the tumor niche.

**Figure 9:**
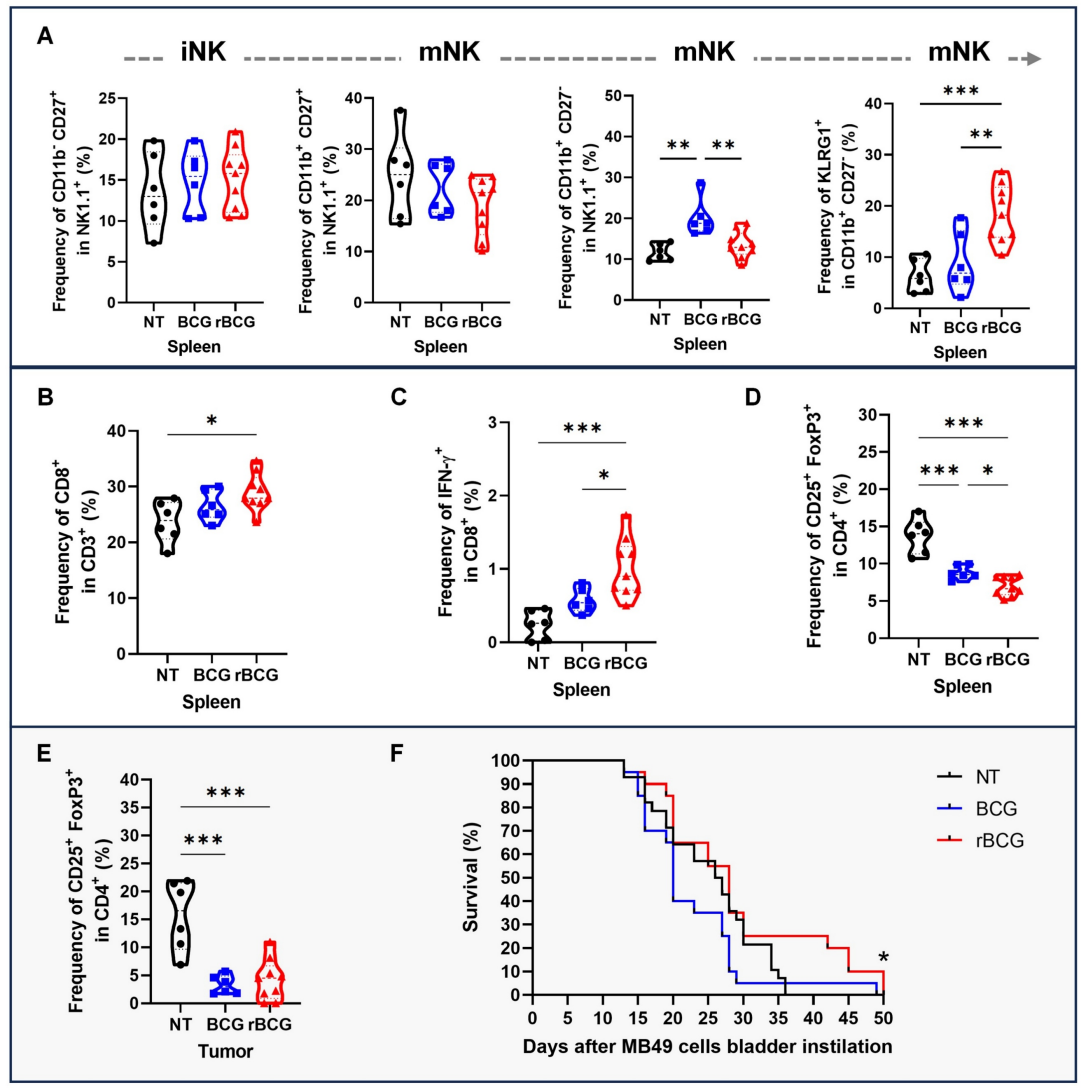
** rBCG enhances NK cells and CD8⁺ T cell activation and mitigates immunosuppression in the tumor microenvironment.** (A) Frequencies in the spleen of NK cell subsets: immature (CD11b⁻ CD27⁺), transitional mature (CD11b⁺ CD27⁺), terminally mature effector (CD11b⁺ CD27⁻), and KLRG1⁺ terminally mature NK cells. (B) Frequency of CD8^+^ population in the spleen. (C) Frequency of IFN-γ expression within the CD8^+^ population in the spleen. (D) Frequency of CD25^+^ FoxP3^+^ population in the spleen. (E) Frequency of CD25^+^ FoxP3^+^ population in the tumor. (F) Kaplan-Meier survival curves of tumor-bearing mice. Statistical significance indicates comparisons between the rBCG group against NT and BCG. NT - no treated; BCG - BCG Danish; rBCG - rBCG-LTAK63. ANOVA statistical analysis (*p < 0.05, **p < 0.01, ***p < 0.001). Data are representative of two independent experiments, with n=10 mice per experimental group.

To further assess effector responses, NK cell maturation and activation were evaluated. rBCG increased the frequency of terminally mature (CD11b⁺ CD27⁻) NK cells and KLRG1⁺ NK cells in the spleen, consistent with a more activated phenotype (Figure 9A). In parallel, rBCG treatment expanded the splenic CD8⁺ T cell population (Figure 9B) and significantly enhanced their functional activity, as shown by increased IFN-γ production (Figure 9C). Analysis of Tregs revealed that rBCG reduced the frequency of CD25⁺ FoxP3⁺ cells both in the spleen (Figure 9D) and in the tumor (Figure 9E), mitigating immunosuppressive mechanisms. Importantly, these immune correlates were associated with improved survival, as Kaplan-Meier analysis demonstrated that rBCG significantly prolonged survival compared to NT and showed a clear advantage over parental BCG (Figure 9F).

Together, these results in the orthotopic bladder cancer model closely resemble those obtained in the heterotopic setting, demonstrating that rBCG consistently outperforms parental BCG in two independent models and further supporting its promise as a superior immunotherapeutic strategy.

## Discussion

Despite being the standard of care for NMIBC, BCG therapy presents significant limitations, particularly due to resistance and therapeutic failure in a subset of patients. These challenges have fueled the search for next-generation BCG formulations, capable of eliciting stronger and more sustained antitumor immune responses. In this context, we present, for the first time, the use of rBCG as a cancer immunotherapeutic, a recombinant BCG strain designed to enhance immunogenicity through the expression of the non-toxic LTK63 subunit, a well-characterized mucosal adjuvant 20. Our findings reveal that rBCG exhibits superior efficacy over parental BCG in controlling tumor growth, both *in vitro* and *in vivo*. This enhanced therapeutic potential was closely linked to a robust activation of innate and adaptive immune cells, particularly dendritic cells, CD4⁺ T cells, and CD8⁺ T cells, which orchestrated a potent antitumor immune response.

The LTK63 has been widely studied regarding the ability to stimulate immune responses by promotion of chemokines and cytokines secretion, facilitating the recruitment of neutrophils, NK cells, macrophages, dendritic cells, B cells, and T cells 20. However, the use of enterotoxin-derived adjuvants requires careful consideration, as safety concerns have historically limited their clinical application. The *Escherichia coli* heat-labile toxin (LT) is a potent AB₅-type enterotoxin with well-defined mucosal adjuvant properties. Genetically detoxified mutants such as LTK63, in which the serine at position 63 of the A subunit is replaced by lysine, were developed to preserve adjuvanticity while abolishing enzymatic ADP-ribosyl transferase activity 21,22. However, clinical trials using LTK63 as an intranasal adjuvant were interrupted due to transient Bell's palsy, attributed to the GM1-binding B subunit that mediates neuronal uptake of the holotoxin 23. In contrast, rBCG-LTAK63 expresses only the genetically detoxified A subunit (LTAK63), which lacks the GM1-binding B subunit and shows markedly reduced ADP-ribosyl transferase activity and cAMP induction. This configuration eliminates the mechanism underlying neurotoxicity, while retaining the ability to enhance antigen-presenting cell activation 13,15. These immune profiles are essential for the generation of effective antitumor immunity.

Initially developed for tuberculosis control, rBCG-LTAK63 demonstrated superior efficacy in eliciting a robust Th1/Th17 response, with increased IFN-γ, TNF-α, IL-2, and IL-17 production compared to conventional BCG 13. These findings were corroborated in both prophylactic 15 and therapeutic applications 17 against tuberculosis in BALB/c mice. In the present study, all *in vivo* and *ex vivo* experiments were conducted in C57BL/6 mice to meet the syngeneic requirements of the MB49 bladder tumor model. Although this strain exhibits a well-known Th1/inflammatory bias, the same studies cited above demonstrated that rBCG-LTAK63 can elicit a predominantly Th1-skewed immune response even in Th2-prone BALB/c mice. Collectively, these data indicate that the therapeutic effects observed in this study are not merely attributable to the C57BL/6 genetic background but rather reflect the intrinsic capacity of rBCG-LTAK63 to promote a robust Th1-oriented immune response.

From a translational perspective, the feasibility of developing rBCG-LTAK63 as a clinical product is supported by advances in recombinant BCG platforms. Our group generated an auxotrophic BCGΔlysA strain expressing LTAK63 using CRISPR/Cas9 technology, ensuring unmarked and genetically stable expression of the adjuvant gene 14. The auxotrophic background provides an additional biosafety layer, as the strain is unable to persist without lysine supplementation, directly addressing regulatory requirements for environmental risk mitigation and genetic stability of live recombinant vaccines. Moreover, the successful clinical advancement of VPM1002, a recombinant BCG carrying listeriolysin O, into Phase III trials demonstrates that large-scale GMP production and regulatory approval of recombinant BCG strains are feasible 24,25. Therefore, these elements indicate that rBCG-LTAK63 has a realistic translational pathway toward clinical development.

Given the well-established immunogenic properties of rBCG-LTAK63 in the tuberculosis model, we hypothesized that this rBCG could also enhance antitumor immunity in bladder cancer. To evaluate this, we performed a proof-of-concept study using a murine model of bladder cancer, comparing its efficacy to parental BCG immunotherapy.

When evaluating the early immune responses, we observed that rBCG triggered significantly stronger pro-inflammatory responses compared to parental BCG, characterized by higher levels of TNF-α, IL-17, and IFN-γ. This led to greater activation of both CD4⁺ and CD8⁺ T cells, which was evident in both 2D and 3D co-culture systems. Since MB49 cells do not express cytokines 26, the elevated cytokine levels observed in our assays can be attributed entirely to the splenic immune cells, underscoring the intrinsic immunostimulatory potential of rBCG. Furthermore, both BCG and rBCG demonstrated direct tumor-suppressive effects in spheroid cultures, but rBCG exhibited superior control when immune cells were present. This emphasizes the importance of immune activation in maximizing the therapeutic efficacy of BCG. Notably, these findings align with prior tuberculosis studies, where rBCG demonstrated enhanced inflammatory responses 13-17,27.

In the heterotopic bladder cancer model, rBCG consistently exhibited greater tumor suppression than parental BCG. However, systemic immune activation required multiple immunotherapy cycles, with CD4⁺ and CD8⁺ T cell activation becoming evident in the bloodstream only after the final treatment cycle, and exclusively in the rBCG-treated group. In contrast, BCG failed to induce systemic T cell activation, showing no difference from the untreated group. This observation mirrors the clinical necessity for repeated BCG instillations in patients, reinforcing the rationale behind current intravesical BCG treatment regimens 28. Indeed, in the orthotopic bladder cancer model, which more closely recapitulates the clinical setting, rBCG again demonstrated superior efficacy compared with parental BCG. Intravesical administration of rBCG significantly reduced tumor burden, as evidenced by macroscopic bladder examination and tumor-to-bladder weight ratios, confirming that rBCG exerts potent antitumor effects across distinct anatomical sites.

The tumor microenvironment ultimately dictates the fate of the host immune system, a process that is fundamental to understanding the dual roles of immune-mediated tumor suppression and tumor-driven immune evasion. Indeed, cancer treatment remains a significant challenge, as both local and systemic tumor-derived factors create a profoundly immunosuppressive environment 29. For instance, peripheral blood monocytes from cancer patients frequently differentiate into immunosuppressive antigen-presenting cells (APCs), where soluble factors condition immune cells to remain functionally impaired against the tumor 30,31. Reversing this immunosuppressive landscape is a key objective of effective immunotherapy, as it can reprogram immune, stromal, and tumor cells back into the elimination phase, restoring efficient antitumor responses. In this regard, a previous study on tuberculosis demonstrated that human M2 macrophages infected with rBCG upregulate genes associated with inflammation (TAP1, GBP1, SLAMF7, TNIP1, and IL6) and induced secretion of pro-inflammatory cytokines and tissue repair factors, including MCP-3 and EGF 27. These findings suggest that rBCG possesses the ability to counteract immunosuppressive mechanisms, fostering enhanced activation of immune cells and more efficient control of *M. tuberculosis* and tumor cells.

Building on the evidence, we propose that the enhanced antitumor potential of rBCG is likely driven by heightened activation of dendritic cells, which in turn enhances MHC-II expression and TNF-α secretion. This effect is further reinforced by an increased frequency of TNF-α and IFN-γ-producing CD4⁺ and CD8⁺ T cells in the spleen, leading to greater activation of intratumoral dendritic cells and cytotoxic CD8⁺ T cells, while simultaneously reducing the frequency of Tregs. Interestingly, the success of BCG therapy is greatly dependent on neutrophil-mediated antigen presentation. Neutrophils play a central role in phagocytosing BCG, migrating to lymphoid organs, and interacting with dendritic cells and NK cells, thereby priming adaptive immune responses 32-35. Beyond their direct phagocytic function, neutrophils are also essential for NK cell maturation, and their dendritic cell-dependent activation is characterized by increased IL-12 production and reduced IL-10 levels, fostering a more immunostimulatory environment 32. Our findings support this concept, suggesting that rBCG amplifies neutrophil-dependent dendritic cell activation, resulting in higher TNF-α production in the spleen and enhanced NK cell activation at the tumor site. Moreover, antigen presentation activity, whether from BCG or tumor antigens, appears to be enhanced in tumor-bearing animals treated with rBCG. These animals exhibit a higher prevalence of Th1 and Tc1 cells (IFN-γ and TNF-α-producing CD8^+^ T cells) in the spleen. Consistently, tumor-bearing animals treated with either BCG or rBCG-LTAK63 displayed increased frequencies of CD8α⁺ dendritic cells in both spleen and tumor compared with untreated controls (data not shown). Although no significant differences were detected between the two vaccinated groups, this pattern supports the occurrence of cross-priming in this model.

In line with these findings, recent work has elegantly demonstrated that engineered bacterial outer membrane vesicles (OMVs) carrying CXCL9 and IL-12 can remodel the tumor microenvironment and enhance antitumor immunity in bladder and breast cancer models, including humanized mice 36. Compared with rBCG, both strategies converge on the principle of boosting local immune activation to improve therapeutic efficacy in NMIBC. Nevertheless, while OMVs constitute a non-replicative, cell-free platform that provides potent but transient immune stimulation, rBCG-LTAK63 represents a live recombinant strain capable of replication and persistent antigen delivery. Furthermore, it is well established that BCG enhances tumor-specific immunity by reducing T cell exhaustion and promoting IFN-γ production, thereby augmenting tumor immunogenicity and facilitating apoptosis 32,37. Our findings suggest that rBCG amplifies this effect, as evidenced by the greater prevalence of IFN-γ⁺ and TNF-α⁺ CD8⁺ T cells in both the spleen and tumor microenvironment, highlighting the potential to drive more effective and sustained antitumor immune responses.

In conclusion, our findings demonstrate that rBCG exhibits superior tumor suppression effects in both *in vitro* and *in vivo* experimental conditions, and the enhanced therapeutic effects were closely linked to robust activation of innate and adaptive immune cells, particularly dendritic cells, CD4⁺ T cells, and CD8⁺ T cells, which orchestrated potent antitumor responses. This cell activation profile was dependent on multiple immunotherapy cycles, reinforcing the need for repeated BCG instillations in clinical settings. Furthermore, rBCG treatment leads to an impressive reduction of Treg frequency and a less immunosuppressive landscape in the tumor microenvironment. These findings establish rBCG as a promising alternative for BCG in the treatment of NMIBC, providing new insights into immune modulation in bladder cancer and potential paradigm shifts in future treatment approaches.

## Supplementary Material

Supplementary figures and table.
