# Supplementary material for: rBCG-LTAK63 Outperforms BCG in Bladder Cancer Immunotherapy: Dendritic and T Cell Coordination Drives Superior Tumor Control in a Mouse Model
Source: Int J Biol Sci. 2026 Jan 1;22(2):713–30. doi: 10.7150/ijbs.118329 (PMC12781081; doi:10.7150/ijbs.118329)
Supplement: Supplementary file 1 — Supplementary figures and table. [file ijbsv22p0713s1.pdf]

Supplementary figures and legends

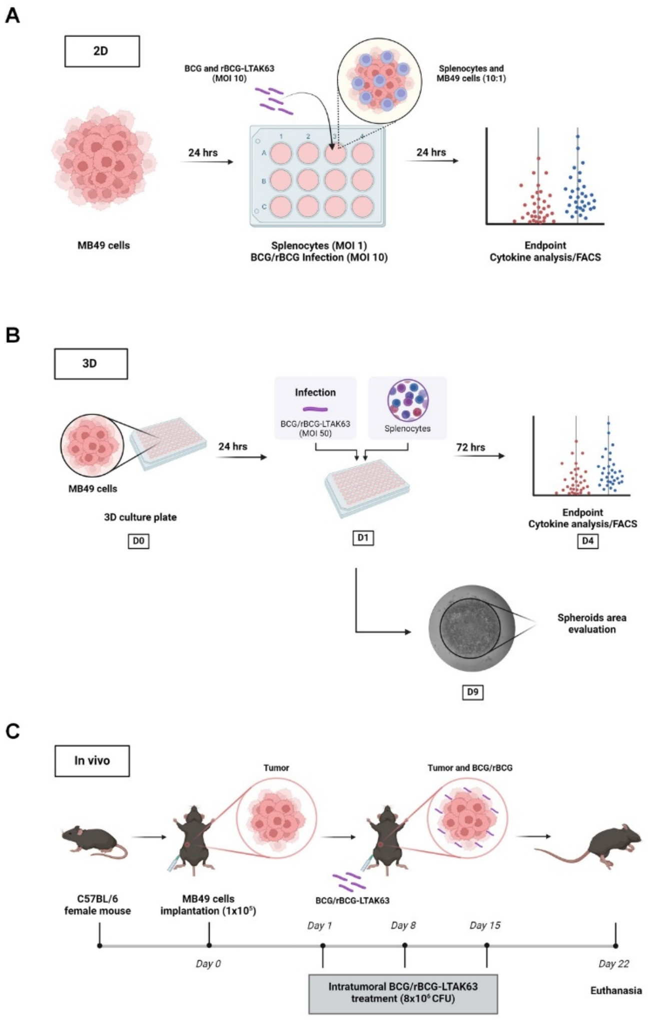

Figure S1. Experimental designs of in vitro and in vivo assays. (A) Bidimensional co-culture (2D): MB49 cells (1×10^5) were seeded in 24-well plates (500 µL/well) on day 0 (D0). After 24 h, medium was replaced with fresh medium containing BCG or rBCG-LTAK63 (MOI 10) and splenocytes from naïve mice (1×10^6; MOI 10), according to the following groups: non-treated (NT; MB49 + splenocytes), BCG (MB49 + splenocytes + BCG), rBCG (MB49 + splenocytes + rBCG-LTAK63), and LPS (50 ng/mL) as a positive control. On D2, supernatants were collected for cytokine analysis (CBA, BD #560485), and immune cells were analyzed by flow cytometry for $CD4^+CD69^+$ and $CD8^+CD69^+$ T cell activation. Antibodies are detailed in Supplementary Table 1. (B) Three-dimensional co-culture (3D): MB49 spheroids (1×10^4 cells/well) were generated in low-attachment 96-well plates and incubated (D0). On D1, BCG or rBCG (MOI 50) and/or splenocytes (MOI 10) were added. LPS (50 ng/mL) was used as a positive control. Spheroids were imaged on D2–D4, and areas were quantified using ImageJ. On D4, supernatants were collected for cytokine profiling (CBA, BD). (C) *In vivo* tumor model and immunotherapy: MB49 cells (5×10^5/100 µL) were injected subcutaneously into the lower right flank of C57BL/6 mice (D0). Immunotherapy was administered intratumorally on D1, D8, and D15 (8×10^6 CFU of BCG or rBCG-LTAK63). On D22, all mice were euthanized, followed by removal of tumors and spleens, which were processed to obtain total immune cells. Groups: NT (tumor only), BCG (tumor + BCG), and rBCG (tumor + rBCG-LTAK63).

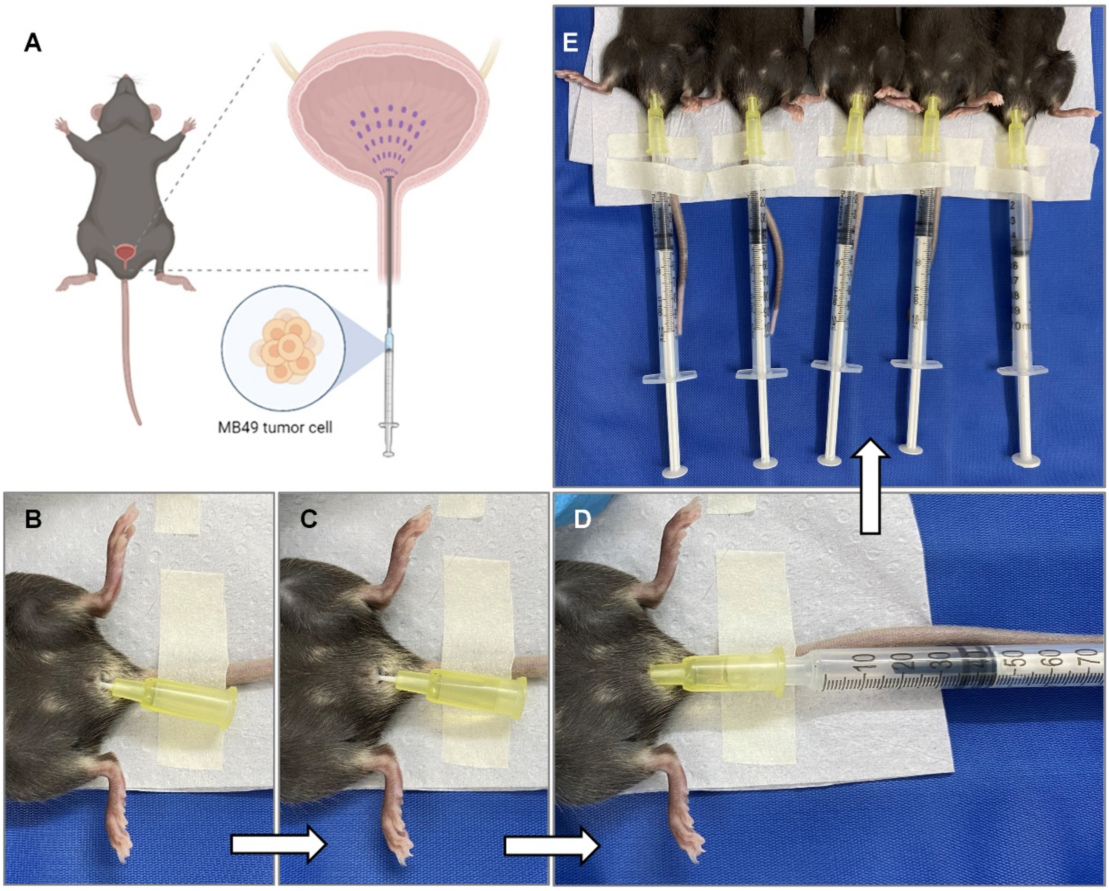

Figure S2. Representative procedure for MB49 tumor cell intravesical instillation in mice. (A) Schematic illustration of the intravesical instillation procedure. (B) Urethral catheterization in an anesthetized mouse. (C) Delivery of tumor cell suspension through the catheter. (D) Instillation of the suspension into the bladder using a syringe connected to the catheter. (E) Maintenance of body temperature on a heating pad throughout the procedure.

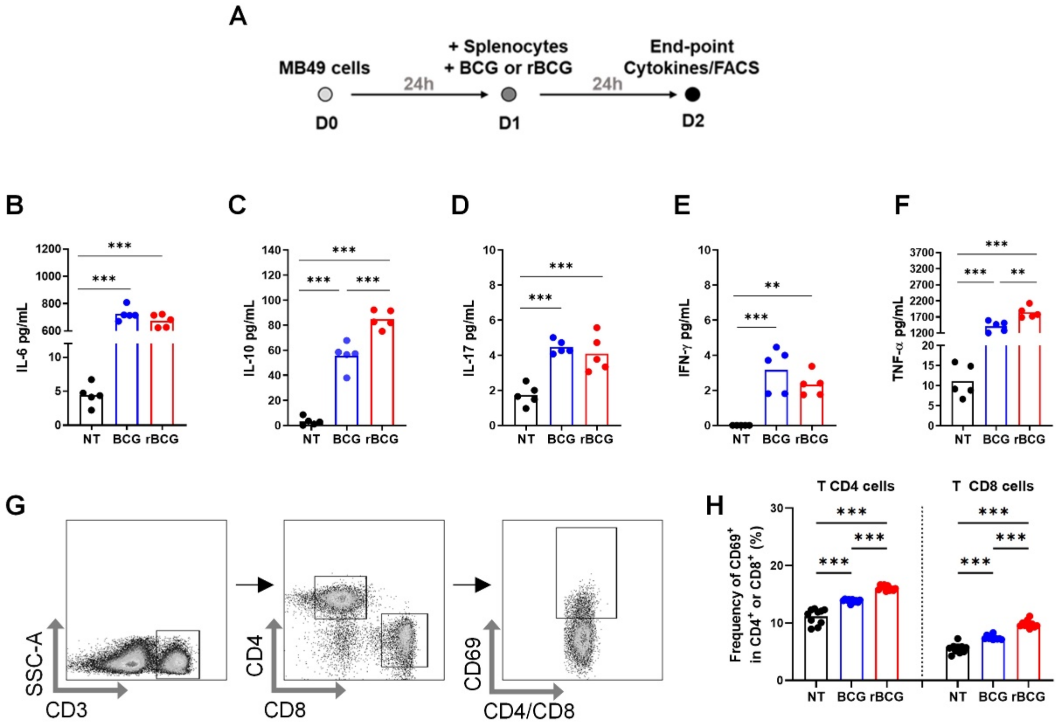

Figure S3. rBCG-LTAK63 promotes higher activation of immune cells in a two-dimensional co-culture of MB49 cells and splenocytes. (A) Timeline of the experimental design. Levels of: IL-6 (B), IL-10 (C), IL-17 (D), IFN-γ (E), TNF-α (F), in the co-culture supernatant. (G) Gate strategy. (H) Frequency of CD69 expression within the CD4 and CD8 T cell populations. ANOVA statistical analysis (**$p < 0.01$, ***$p < 0.001$).

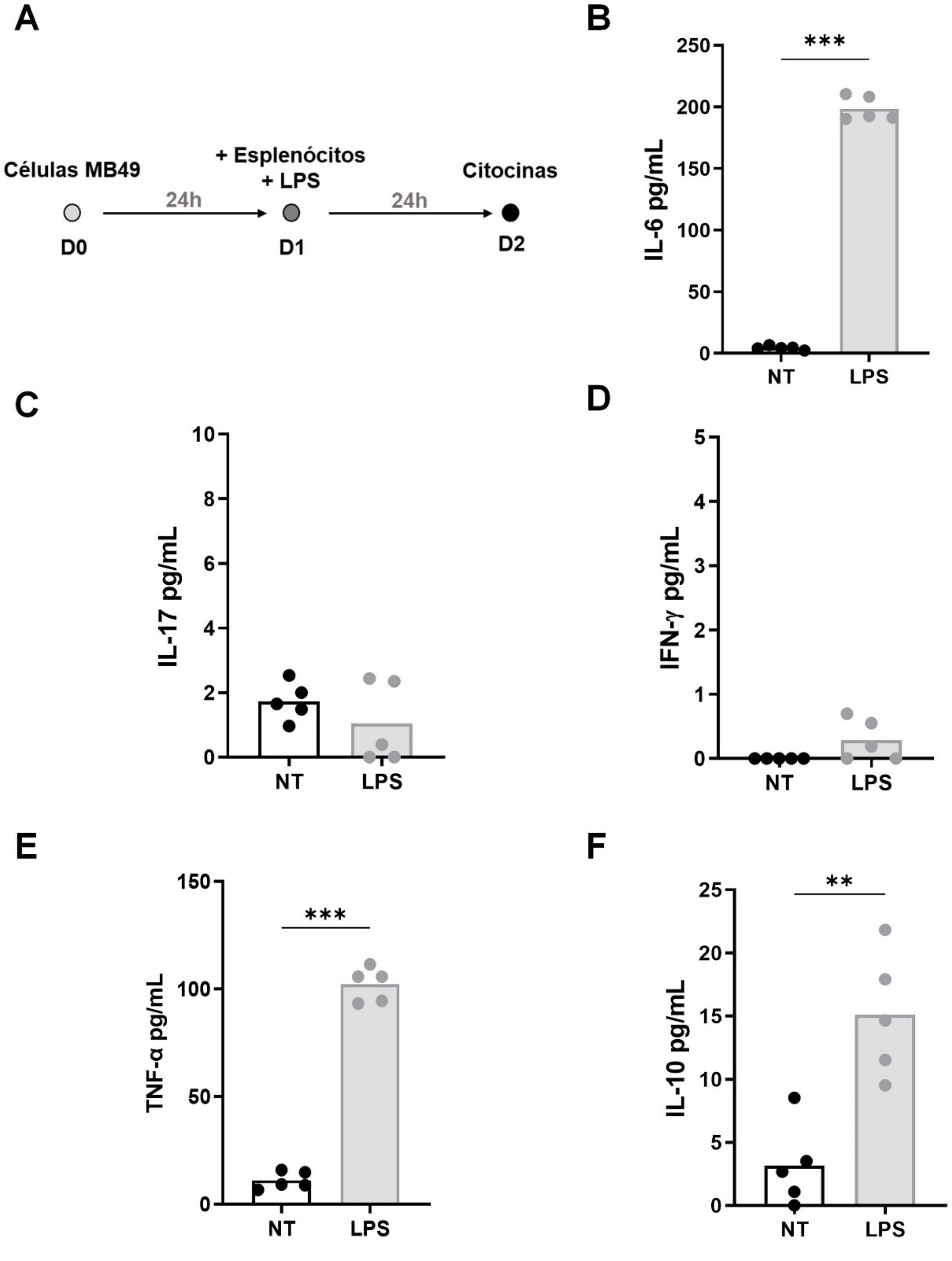

Figure S4. LPS promotes higher activation of immune cells in a two-dimensional co-culture of MB49 cells and splenocytes. (A) Timeline of the experimental design. Levels of: IL-6 (B), IL-10 (C), IL-17 (D), IFN-γ (E), TNF-α (F), in the co-culture supernatant. ANOVA statistical analysis (**$p < 0.01$, ***$p < 0.001$).

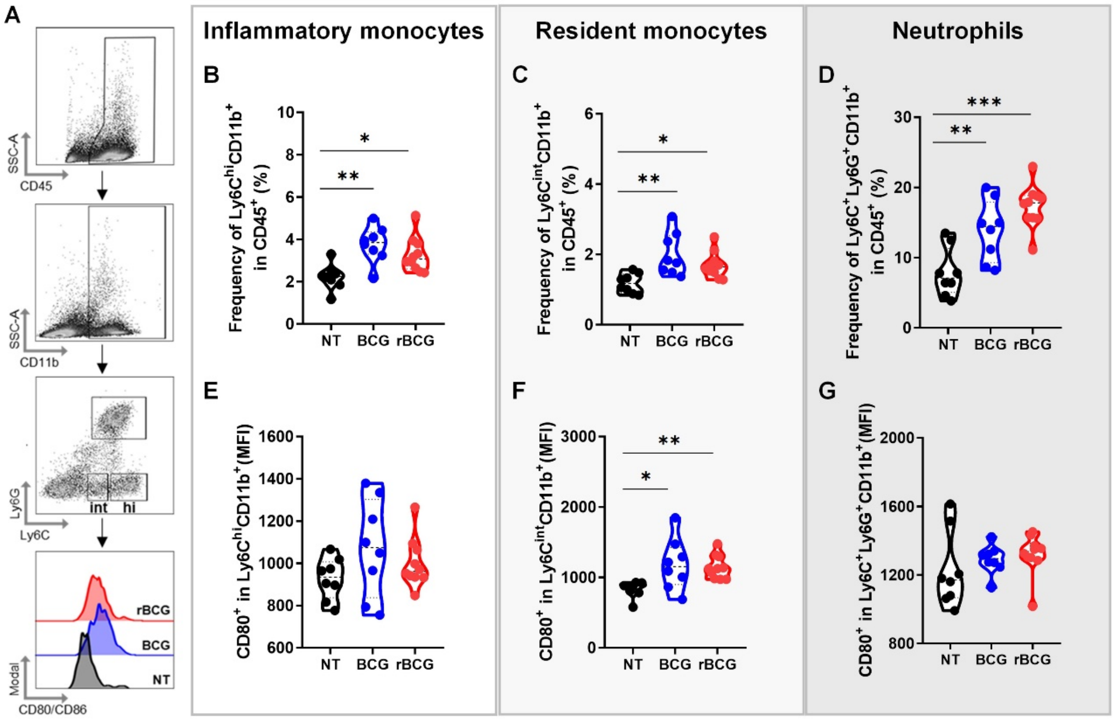

Figure S5. Treatment with BCG and rBCG induces increased recruitment of monocytes and neutrophils to the spleen. (A) Gating strategy for myeloid cell panel. CD45-expressing cells were selected from the previous single-cell analysis. (B) Frequency of Ly6Chi Ly6G- CD11b+. (C) Frequency of Ly6Cint Ly6G- CD11b+ population. (D) Frequency of Ly6C+ Ly6G+ CD11b+ population. (E) Median fluorescence of CD80 within the Ly6Chi Ly6G- CD11b+ population. (F) Median fluorescence of CD80 within the Ly6Cint Ly6G- CD11b+ population. (G) Median fluorescence of CD80 within the Ly6C+ Ly6G+ CD11b+ population. NT - no treated; BCG – BCG Danish; rBCG – rBCG-LTAK63. ANOVA statistical analysis (*$p < 0.05$, **$p < 0.01$, ***$p < 0.001$).

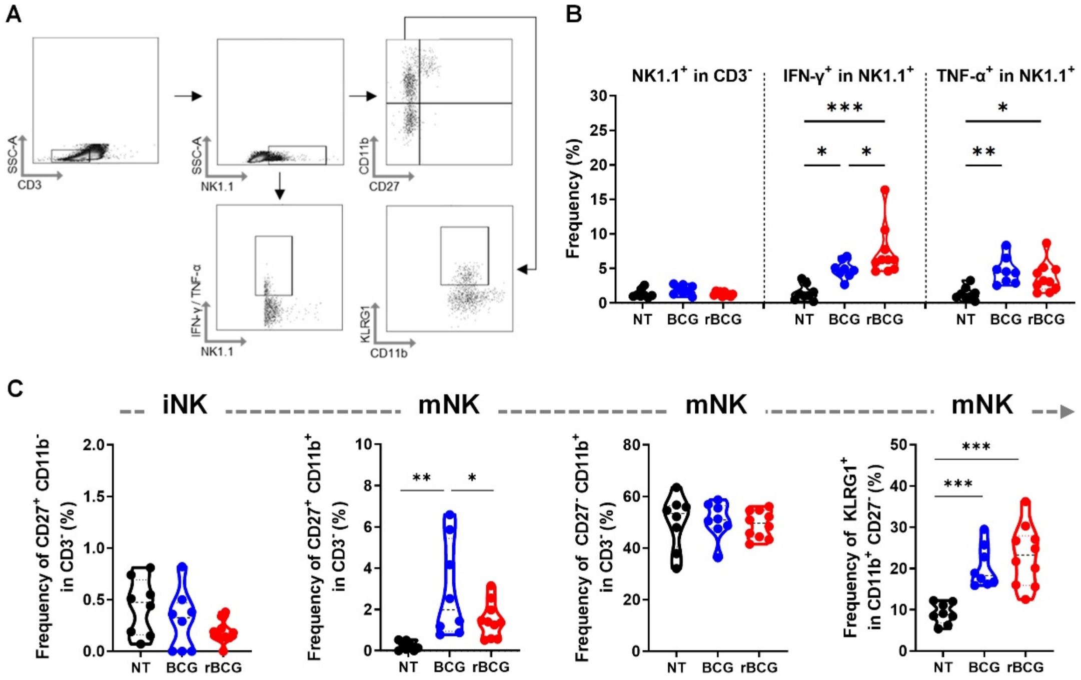

Figure S6. Enhanced splenic NK Cell functionality and maturation following BCG and rBCG immunotherapy. (A) Gating strategy for NK cell panel. (B) Frequency of NK1.1^{+} population; Frequency of IFN-γ expression within the NK1.1^{+} population; Frequency of TNF-α expression within the NK1.1^{+} population. C) iNK - Frequency of CD27^{+} CD11b^{-} population; mNK (inflammatory NK cells) - Frequency of CD27^{+} CD11b^{+} population; mNK - Frequency of CD27^{-} CD11b^{+} population; mNK - Frequency of KLRG1^{+} within CD27^{-} CD11b^{+} population. NT - no treated; BCG – BCG Danish; rBCG – rBCG-LTAK63. ANOVA statistical analysis (*$p < 0.05$, **$p < 0.01$, ***$p < 0.001$).

Supplementary Table

Supplemental table 1: Immunophenotyping panel.

Immunologic cells	Immunophenotyping markers	Activation markers
Lymphocytes T CD4	$CD45^+$, $CD3^+$, $CD4^+$	$CD69^+$, IFN-g, TNF-α
Lymphocytes T CD8	$CD45^+$, $CD3^+$, $CD8^+$	$CD69^+$, IFN-g, TNF-α
Regulatory T cells	$CD45^+$, $CD3^+$, $CD4^+$, $CD25^+$, $FoxP3^+$	-
NK cells	$CD45^+$, $CD3^-$, $NK1.1^+$	IFN-g, TNF-α
Immature NK cells	$CD45^+$, $CD3^-$, $NK1.1^+$, $CD27^+$, $CD11b^-$	-
Inflammatory NK cells	$CD45^+$, $CD3^-$, $NK1.1^+$, $CD27^+$, $CD11b^+$	IFN-g, TNF-α
Mature NK cells I	$CD45^+$, $CD3^-$, $NK1.1^+$, $CD27^-$, $CD11b^+$	IFN-g, TNF-α
Mature NK cells II	$CD45^+$, $CD3^-$, $NK1.1^+$, $CD27^-$, $CD11b^+$	$KLRG1^+$
Dendritics cells	$CD45^+$, $CD11c^+$, $MHC\text{-}II^+$	$CD80^+$, $CD86^+$, TNF-α
Macrophages	$CD45^+$, $CD11b^+$, $F4/80^+$	$CD80^+$, $CD86^+$, TNF-α
Inflammatory monocytes	$CD45^+$, $CD11b^+$, $Ly6C^{hi}$, $Ly6G^-$	$CD80^+$, $CD86^+$, TNF-α
Resident monocytes	$CD45^+$, $CD11b^+$, $Ly6C^{int}$, $Ly6G^-$	$CD80^+$, $CD86^+$, TNF-α
Neutrophils	$CD45^+$, $CD11b^+$, $Ly6C^+$, $Ly6G^+$	$CD80^+$, $CD86^+$, TNF-α
